# A Novel Method to Identify the Differences Between Two Single Cell Groups at Single Gene, Gene Pair, and Gene Module Levels

**DOI:** 10.3389/fgene.2021.648898

**Published:** 2021-03-15

**Authors:** Lingyu Cui, Bo Wang, Changjing Ren, Ailan Wang, Hong An, Wei Liang

**Affiliations:** ^1^School of Science, Dalian Maritime University, Dalian, China; ^2^Geneis (Beijing) Co., Ltd., Beijing, China; ^3^Guangzhou Anjie Biomedical Technology Co., Ltd., Guangzhou, China; ^4^Medical Clinical Laboratory, The Second People's Hospital of Lianyungang, Lianyungang, China

**Keywords:** scRNA-seq, differential gene expression analysis, differential correlation analysis, network analysis, differential network analysis

## Abstract

Single-cell sequencing technology can not only view the heterogeneity of cells from a molecular perspective, but also discover new cell types. Although there are many effective methods on dropout imputation, cell clustering, and lineage reconstruction based on single cell RNA sequencing (RNA-seq) data, there is no systemic pipeline on how to compare two single cell clusters at the molecular level. In the study, we present a novel pipeline on comparing two single cell clusters, including calling differential gene expression, coexpression network modules, and so on. The pipeline could reveal mechanisms behind the biological difference between cell clusters and cell types, and identify cell type specific molecular mechanisms. We applied the pipeline to two famous single-cell databases, Usoskin from mouse brain and Xin from human pancreas, which contained 622 and 1,600 cells, respectively, both of which were composed of four types of cells. As a result, we identified many significant differential genes, differential gene coexpression and network modules among the cell clusters, which confirmed that different cell clusters might perform different functions.

## Introduction

The fundamental unit of an organism is the cell. Coordinated gene expression in each cell is essential to biological functions, and aberrations often cause illness. Consequently, the genome-wide quantification of RNA experiments help to understand the growth and development of organism as well as pathogenesis of disease. One traditional technology of mRNA abundance measured at cell line or tissue level averaged over thousands or millions of cells, which is also called bulk RNA-seq (Stark et al., 2019). The bulk RNA-seq experiments has been successfully applied to a multitude of studies, and improved our biology knowledge. However, the disadvantage of bulk RNA-seq is that cell-specific mRNA abundance could not been provided, and some important gene expression signals might be unobserved. Our current knowledge related with cell types and there dynamic changes in biological system remains highly incomplete. Owing to resolution in sequencing technology, single-cell RNA-seq (scRNA-seq) at genome-wide level was first invented by Tang et al. (2009), and has been under rapidly booming development. The scRNA-seq technology makes some very important and challenging scientific research possible. For instance, unknown cell types were identified (Trombetta et al., 2014; Buettner et al., 2015). How to dissect gene expression changes during dynamic development (Tang et al., 2010; Xue et al., 2013; Yan et al., 2013). Study uncovered how tumorgenesis and cancer cell immune escape and tumor cell heterogeneity (Chung et al., 2017; Zhao et al., 2020). scRNA-seq was also used to predict therapeutic response in patients and understanding drug resistance mechanism (Lee et al., 2014; Liang et al., 2020), and clarify the pathophysiology of complex diseases and guide the successful treatment and intervention of patients with intractable diseases (Shalek and Benson, 2017; Kim et al., 2020). Collectively, the scRNA-seq technology has significantly promoted basic biological research and clinical personalized medicine. At the same time, the analysis of scRNA-seq data is challenging due to a number of problems such as sparsity caused by technical dropout, bimodal and multi-modal expression distributions (Korthauer et al., 2016), and highly biological and technical cell-to-cell variability (Vallejos et al., 2017; Hicks et al., 2018) giving rise to cellular heterogeneity. One very important step of scRNA-seq data analysis is to identify gene-specific expression pattern and/or a gene-gene interacting network within a population of cells or a biological condition in studies. Although numerous computational methods have been developed and applied during the past few years, most of them focused on difference in single gene-level differentiation (Finak et al., 2015; Korthauer et al., 2016; Butler et al., 2018; Miao et al., 2018; Stuart et al., 2019). In the present study, we integrated a variety of computational methods into a variance analysis workflow.

A fundamental question raised of expression data is what genes differentially expressed across conditions and circumstances. Despite technological revolution for scRNA-seq in recent years, technical stability of RNA quantification by scRNA-seq is still worse than that in bulk RNA-seq. Thus, the numerous variation computational tool established for bulk RNA do not work well for single-cell RNA-seq. During the past few years, a couple of computational methods have been designed particularly for single-cell RNA-seq data (Soneson and Robinson, 2018). For example, MAST based on Generalized linear model (Finak et al., 2015); DEsingle based on Zero inflated negative binomial (Miao et al., 2018); D3E based on Cramér-von Mises test, Kolmogorov-Smirnov test, likelihood ratio test (Delmans and Hemberg, 2016); SCDE based on Poisson and negative binomial model (Kharchenko et al., 2014); SigEMD based on Non-parametric earth mover's distance (Wang and Nabavi, 2018) and so on. Marker genes found by differential expression analysis play important role in cell type identification and discovery. It is also essential for downstream drug targets prediction and thus to prevent or treat disease. In addition to analyzing single gene, analyzing the relationship between genes is also crucial for construction of biological networks. For instance, the R package DGCA offers a suite of tools for computing and analyzing differential correlations between genes across multiple conditions (McKenzie et al., 2016).

If some genes always have similar expression patterns in a physiological process or metabolic process, then we can consider these genes to be functionally dependency, so they can be defined as a functional module. If a gene module is identified, then numerous researches would be done based of which, such as screening the core genes of relevant trait modules, modeling metabolic pathways, and establishing gene interaction networks. Weighted correlation network analysis (WGCNA) is a typical analysis tool at the network co-expression level (Langfelder and Horvath, 2008). Since WGCNA is an analysis tool designed for bulk sequencing data, almost no one uses it to analyze scRNA-seq data. Based the correlation between the analyzed module and the sample characteristics we can quickly extract gene co-expression modules related to the sample characteristics from the complex data for subsequent analysis. WGCNA builds a bridge between sample characteristics and gene expression changes (Iancu et al., 2012; Xue et al., 2013).

In the present study, we performed differential expression genes (DEGs) analysis for each two categories in the scRNA-seq data from a single gene level. Based the level of gene pairs, differential correlation analysis for each two categories were analyzed for the purpose of digging deeper biological information. The gene pair with the most significant difference in each category pair was obtained. The results of this analysis provide theoretical support for medical staff. Based the level of gene network, we used WGCNA to perform network analysis on scRNA-seq data, and in order to explore the difference in gene expression of each module, we used DiffCoEx (Tesson et al., 2010) to analyze the difference network module. The results of analysis from different levels of single cells, cell pairs, and cell networks showed that such a complete system is more capable of mining the underlying information contained in the scRNA-seq data. The study provided a comprehensive analysis approach for scRNA-seq researches in future.

## Materials and Methods

**Figure 1:**
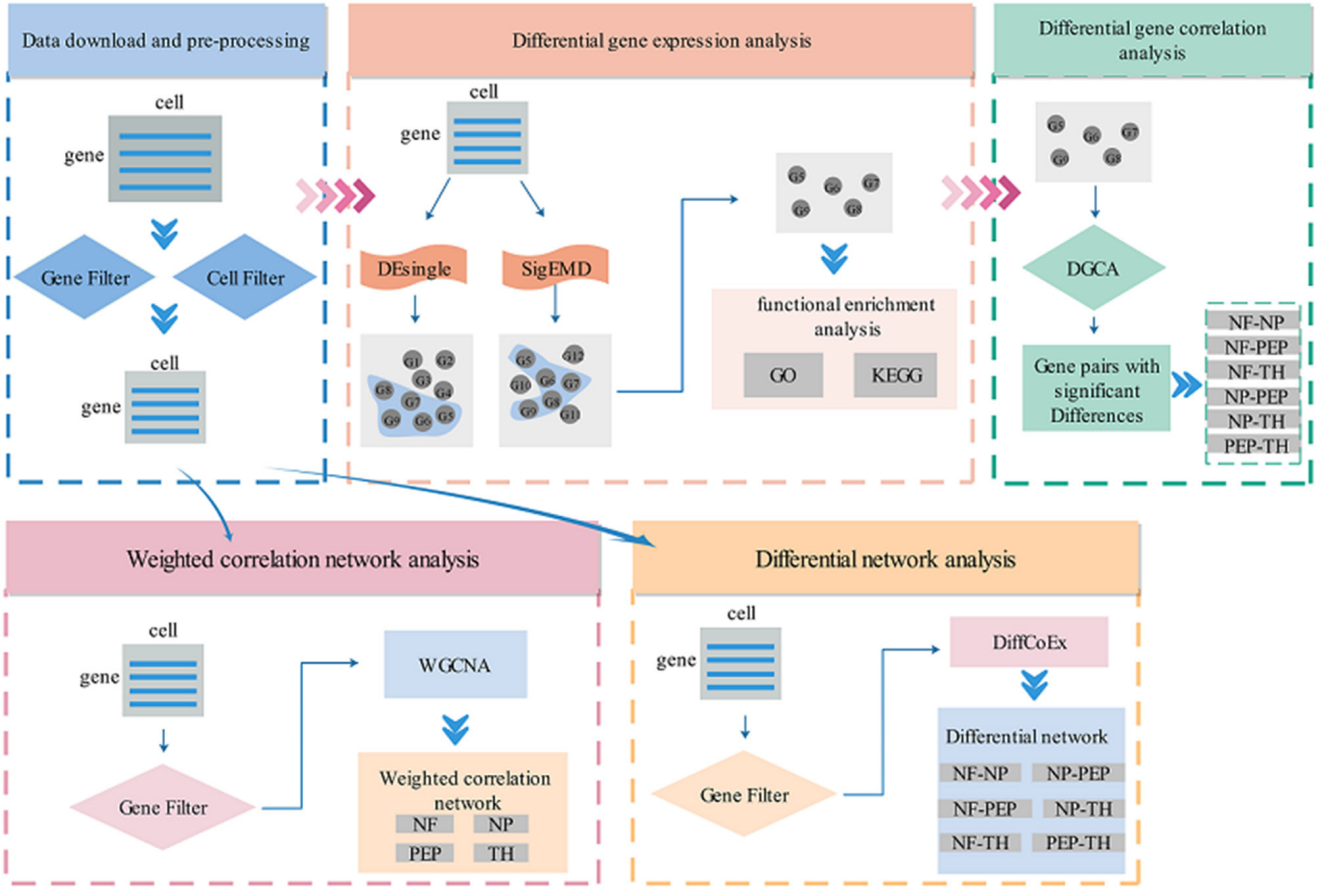
Flow chart of scRNA-seq data analysis. Cat. is the abbreviation of category, cat.1–4, respectively represent four cell types. Note that the gene filtering method in each method is different, please refer to the specific introduction in each section for details.

In recent years, with the microfluidic technology that can separate individual cells from a piece of tissue, researchers have made it more accurate to predict the diversity of biological tissues and target drugs for related diseases. Compared with bulk sequencing technology, the resolution of scRNA-seq technology is very accurate for single-cell level analysis, so scRNA-seq technology has developed rapidly. Although a series of work on single-cell sequencing technology has been developed in recent years, most of them are tested and verified in a single field, and there is no complete system to mine the potentially valuable information in single-cell data. Ignore some algorithms that have been developed in bulk sequencing technology, such as WGCNA. In this work, we have established a set of procedures for analyzing scRNA-seq data, including differential gene expression analysis (DEsingle, SigEMD), differential correlation analysis (DGCA), network analysis (WGCNA), differential network analysis (DNA). The specific flow chart is shown in Figure 1. These processes are described in detail below.

### Data Information

In this work, we used two single-cell data sets. One of them is Usoskin [622 (cells) ^*^ 25335 (genes)], which comes from the GEO database (GSE59739) (Usoskin et al., 2015). This data is mainly divided into 4 categories: NF, NP, PEP, and TH. We performed a concise preprocessing of the data, the gene filter removes genes/transcripts that are expressed in <3 cells, and the cell filter removes cells that are expressed in <500 genes, the number of remaining samples is 622, and the gene dimension is 25333. Table 1 summarizes the basic information of Usoskin data.

**Table 1:** Brief information about Usoskin data.

**Usoskin (622)**	**Num. of cells**	**Num. of genes**	**Description of cell groups**
NF	139	25333	Neurofilament containing
NP	169		Non-peptidergic nociceptors
PEP	81		Peptidergic nociceptors
TH	233		Tyrosine hydroxylase containing

The other data used in this article is from human pancreas, named Xin [1600 (cells) ^*^ 39851 (genes)], which comes from the GEO database (GSE81608) (Xin et al., 2016). Xin is also divided into four categories: α, β, δ, and PP. The data uses the same preprocessing method as Usoskin data, the number of remaining samples is 1492, and the gene dimension is 28403. Table 2 summarizes the basic information of Xin data.

**Table 2:** Brief information about Xin data.

**Xin (1492)**	**Num. of cells**	**Num. of genes**	**Description of cell groups**
α cells	886	28403	Produce glucagon
β cells	472		Insulin
δ cells	49		Somatostatin
PP cells	85		Pancreatic polypeptide

### Differential Gene Expression Analysis

We performed pairwise difference expression genes (DEG) analysis on the four types of cells in these two data sets. The methods used for DEGs are DEsingle (Miao et al., 2018) and SigEMD (Wang and Nabavi, 2018), both of which are methods for scRNA-seq data.

One of the biggest features of scRNA-seq data is that it contains a high proportion of 0 values, which is mainly due to two reasons: on the one hand, these “true” 0 values are the natural expression values of genes; on the other hand, due to the reverse transcription and sequencing process, there are too many “false” 0 values caused by the technical noise of the company, we call the latter “dropout.” In response to this phenomenon, most of the current differential analysis methods cannot separate the two situations, so DEsingle was developed to solve the differential analysis that contains the dropout problem data. DEsingle employed Zero-Inflated Negative Binomial model to estimate the proportion of real and dropout zeros and detect three types of DEGs in scRNA-seq data with higher accuracy.

The SigEMD method also takes into account the “dropout” problem. Using a logistic regression model and a non-parametric method based on the distance of the earth mover can accurately and effectively identify the DEGs in the scRNA-seq data. Regression models and data imputation are used to reduce the impact of a large number of zero counts, and non-parametric methods are used to improve the sensitivity of detecting DEGs from multimodal scRNAseq data. And used simulated data sets and real data sets to verify the accuracy of this method.

### Differential Gene Correlation Analysis

The key step to establish a biological system prediction model is to analyze the regulatory relationship between genes, so an effective solution is to study the difference in correlation between gene pairs. Differential Gene Correlation Analysis (DGCA) is proposed to solve such problems (McKenzie et al., 2016). In order to minimize parameter assumptions, DGCA calculates empirical *p*-values through permutation tests. In order to understand the differential correlation at the system level, DGCA conducted a higher-level analysis through simulation research. The simple method based on Z score adopted by DGCA is significantly better than the existing alternative methods of calculating differential correlation.

### Network Analysis

Weighted correlation network analysis (WGCNA) is a systems biology method used to describe gene association patterns between different samples (Liu et al., 2017). WGCNA can be used to identify highly coordinated gene sets, and identify candidate biomarker genes or therapeutic targets based on the interconnectivity of gene sets and the association between gene sets and phenotypes. Compared with only focusing on differentially expressed genes, WGCNA uses the information of thousands or tens of thousands of genes with the greatest changes or all genes to identify the gene set of interest, and conducts significant association analysis with the phenotype. It not only makes full use of information, but also converts the associations between thousands of genes and phenotypes into associations between multiple genomes and phenotypes, eliminating the problems of multiple hypothesis testing and correction.

### Differential Network Analysis

In scRNA-seq data, if certain genes always have similar expression changes in a physiological process or in different tissues, then we have reason to believe that these genes are functionally related and can be defined as a module. When the gene module is defined, we can use these results to do a lot of further work. For example, we use DiffCoEx for differential network analysis (Tesson et al., 2010), which is a method for identifying changes in association patterns. This method is based on the commonly used WGCNA framework for co-expression analysis. Prove its usefulness by identifying biologically relevant, differentially co-expressed modules in the mouse dataset.

## Software Availability

The codes for the two methods of differential gene expression analysis are freely available (DEsingle: https://bioconductor.org/packages/DEsingle, SigEMD: https://github.com/NabaviLab/SigEMD); This article uses the DAVID website for feature enrichment analysis. The website is available for free in https://david.ncifcrf.gov/ Difference correlation analysis is freely available in https://github.com/andymckenzie/DGCA WGCNA is freely available in https://cran.r-project.org/web/packages/WGCNA/index.html DiffCoEx is freely available in https://bmcbioinformatics.biomedcentral.com/articles/10.1186/1471-2105-11-497.

## Results

### DEGs Between Two Categories

With the development of high-throughput technology, the field of biomedical related research has entered the omics era, and the research of a single gene can no longer meet the needs of researchers. However, such a large amount of data brings new challenges to the effective extraction and analysis of information. Taking sequencing data as an example, the analysis of sequencing results often results in a list of differentially expressed genes or proteins. But for many researchers, it is difficult to associate this long list of genes or proteins with a biological phenomenon to be studied and its underlying mechanism. Functional enrichment analysis is to divide a gene or protein list into multiple parts, that is, to classify a bunch of genes, and the classification criteria here are often limited according to the function of the gene. In other words, it is to put together genes with similar functions in a gene list and associate them with biological phenotypes.

**Figure 2:**
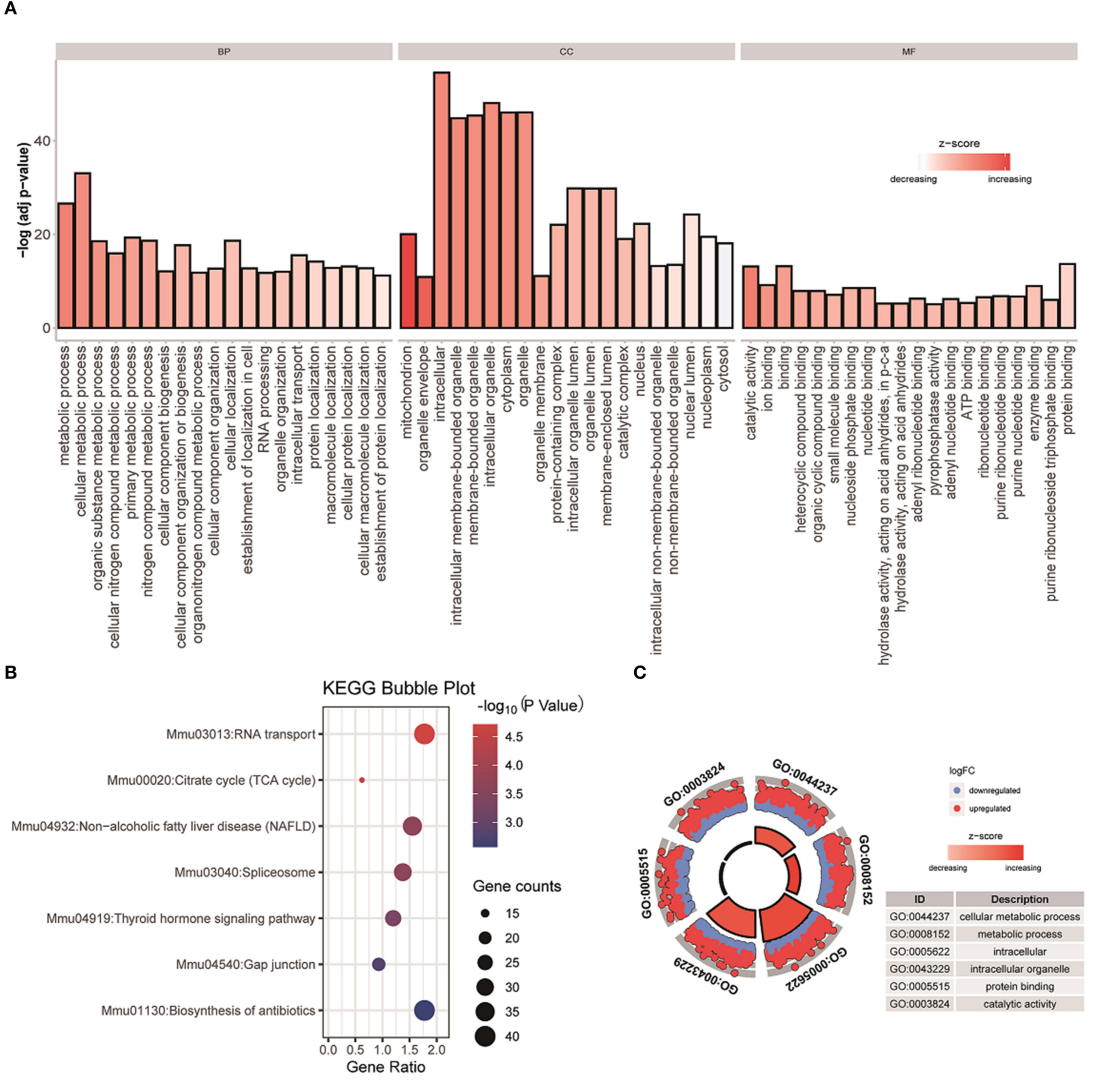
GO and KEGG analysis were performed on the differential genes with overlapping NF-NP data. **(A)** Perform enrichment analysis on the differential genes with overlapping datasets, and display the top 20 most significant terms in BP, CC, and MF. **(B)** Perform KEGG enrichment analysis on the differential genes with overlapping NF-NP data. **(C)** Basic information of six specified terms, among them, blue means down-regulated genes, red means up-regulated genes.

We use DEsingle and SigEMD two methods to analyze the four types of data contained in Usoskin, overlap the differential genes obtained by the two methods, and select the differential genes with *p* < 0.05 for functional enrichment analysis. In this work, we used DAVID to perform two enrichment analyses of GO and KEGG on overlapping differential genes obtained from two NF-NP data, and correlated them with biological phenotypes. Among them, GO (Gene Ontology) enrichment analysis is mainly divided into three parts: Molecular Function (MF), Biological Process (BP), and Cellular Component (CC), as shown in Figure 2A, we have selected the top 20 representative Go terms for BP, CC, and MF. The x-axis represents the first 20 terms selected for each part, the y-axis represents the change of *p*value, and the color represents z-score. The KEGG (Kyoto Encyclopedia of Genes and Genomes) is a database that systematically analyzes the metabolic pathways of gene products in cells and the functions of these gene products. KEGG integrates data on the genome, chemical molecules, and biochemical systems, including metabolic pathways (PATHWAY), etc. As shown in Figure 2B, we can observe that seven pathways are obtained in the two sets of NF-NP data, and the number of genes expressed in the pathway Mmu030133: RNA transport pathway is large, and the *p*-value lower, indicating that the enrichment of this pathway is the most significant. In addition, we selected two terms with the most significant enrichment among the three indicators of BP, CC, and MF, and analyzed the up-regulated and down-regulated genes of these six terms, as well as their z-score changes, as shown in Figure 2C. The Xin data set and the other five analysis results are shown in Supplementary Material 1.

### Gene Pairs With Significant Differences Between Two Categories

Analyzing the regulatory relationship between genes is a key step in establishing an accurate prediction model of biological systems. To achieve this goal, a powerful method is to systematically study the correlation differences between gene pairs in more than one situation. In our work, we will perform pairwise analysis on the four data types contained in Usoskin and Xin, and consider the difference and correlation between gene pairs in different types of datasets. We used overlapping differentially expressed genes as the input of DGCA, and listed the most different gene pairs in six different situations, as shown in Tables 3, 4.

**Table 3:** The six gene pairs in Usoskin data have the largest differences in different situations.

	**Gene1**	**Gene2**	**class1_cor**	**class2_cor**	**zScoreDiff**	**empPVals**	**Classes**
NF_NP	Robo1	Grid1	−0.0797	0.9913	23.5578	3.11102E-08	0/+
NF_PEP	Tomm22	Zc3h13	−0.1886	0.9901	20.0313	3.96294E-08	−/+
NF_TH	Fam84a	Omg	−0.0701	0.9953	25.0786	1.57764E-08	0/+
NP_PEP	Itga3	Synj2	−0.0263	0.9968	19.5367	4.84097E-06	0/+
NP_TH	Il17rd	Pde1b	0.9865	−0.0098	−24.5847	1.25299E-06	+/0
PEP_TH	H2.M11	Pde8b	−0.0716	0.9945	20.6581	2.66991E-07	0/+

**Table 4:** The six gene pairs in Xin data have the largest differences in different situations.

	**Gene1**	**Gene2**	**class1_cor**	**class2_cor**	**zScoreDiff**	**empPVals**	**Classes**
α_β	DAPL1	HMOX1	0.9962	−0.0402	−47.0484	9.33E-09	+/0
α_δ	GCG	G6PC2	−0.1662	0.9967	18.8006	3.79E-07	−/+
α_pp	SLC25A53	RPL518	−0.0630	0.9901	23.6008	6.44E-08	0/+
β_δ	INS	IAPP	−0.1771	0.1000	18.4667	1.33E-06	−/+
β_pp	INS	IAPP	−0.1771	0.1000	23.7250	7.50E-08	−/+
δ_pp	RBP4	SST	−0.0414	0.9979	14.5204	1.53E-05	0/+

The first column in Table 3 shows the matching analysis pairs of six different data subtypes, corresponding to class1 and class2, respectively in columns four and five, and the sixth column shows the change value of Z-score, indicating the change of correlation between gene pairs. Table 4 is the same. NF_NP, NF_PEP, NF_TH, NP_PEP, PEP_TH these five pairs of data from class1 to class2 gene pair correlation completely lost, on the contrary, NP_TH this pair of data is completely irrelevant from class1 to class2 correlation has been significantly improved. Please refer to Table 5 for basic information about the two genes *Il17rd* and *Pde1b*. For detailed information about these two genes, please refer to the database MGI (Mouse Genome Informatics, http://www.informatics.jax.org/).

**Table 5:** Basic information of genes *Il17rd* and *Pde1b*.

	**Il17rd**	**Pde1b**
Name	Interleukin 17 receptor D	Phosphodiesterase 1B, Ca2+-calmodulin dependent
Feature type	Protein coding gene	Protein coding gene
Human ortholog	IL17RD, interleukin 17 receptor D	PDE1B, phosphodiesterase 1B
Chr location	3p14.3; chr3:57089982-57170317 (−) GRCh38.p7	12q13.2; chr12:54549393-54579239 (+) GRCh38.p7
HomoloGene	Vertebrate Homology Class 9717	Vertebrate Homology Class 37370
HCOP	Human homology predictions: IL17RD	Human homology predictions: PDE1B

### Co-expression Networks Generated With WGCNA

WGCNA is mainly divided into two steps. In the first step, WGCNA analysis uses the weighted value of the correlation coefficient, that is, the gene correlation coefficient is taken to the power of β, so that the connection between the genes in the network obeys the scale-free network distribution (scale-free networks), determine the β parameter by the square of the correlation coefficient of log(*k*) and *logp*[(*k*)]. In general, the higher the square of the correlation coefficient, the closer the network is to the distribution without network scale. This algorithm has more biological significance. The second step is to construct a hierarchical clustering tree through the correlation coefficients between genes. Different branches of the clustering tree represent different gene modules, and different colors represent different modules. Based on the weighted correlation coefficients of genes, genes are classified according to their expression patterns, and genes with similar patterns are grouped into one module. In this way, tens of thousands of genes can be divided into dozens of modules through gene expression patterns, which is a process of extracting general information.

**Figure 3:**
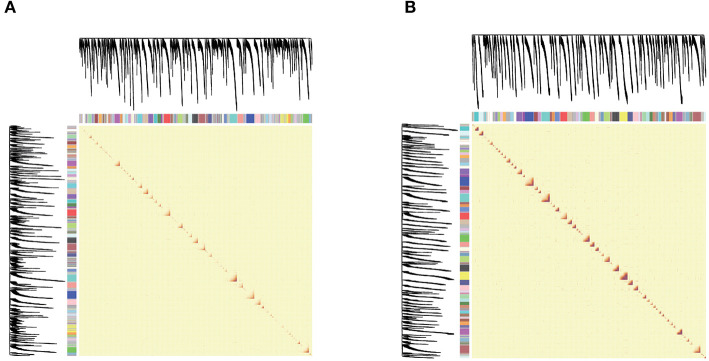
**(A,B)** are network heat maps of NF and PEP, respectively. On the left side and top are the hierarchical clustering trees and modules of genes. In the figure, red represents higher similarity and yellow represents lower similarity. As the module is composed of genes with high similarity, it corresponds to the diagonal red in the figure.

In this work, in order to reduce the running time of WGCNA, we calculated the standard deviation of the genes in each data set, and then left the genes with the largest standard deviation of the first 5000. The reason is that data with large variance contains the main biological information in the data, and it can also reduce the complexity of calculation. We first analyze two data subsets of NF and PEP, as shown in Figure 3. The Xin data set and the other two analysis results are shown in Supplementary Material 2.

Figure 3 shows the heat map of the module. Both the abscissa and the ordinate are genes, and the entire module represents the relationship between genes. On the left and top is the hierarchical clustering tree and module allocation. Red represents higher similarity, and yellow represents lower similarity. Since the module is composed of genes with high similarity, corresponding to the red area of the diagonal line in the figure, the target gene analysis and the correlation between the module and the trait can be performed for the module of interest.

Hub gene is a gene that plays a vital role in biological processes. In related pathways, the regulation of other genes is often affected by this gene. Therefore, hub gene is often an important target and research hot spot. We use *chooseTopHubInEachModule* in the WGCNA package to find the Hub genes in each module, and predict the gene function of the module through functional enrichment analysis. Here we show the Hub genes of the NF data type in Table 6 and the results of the functional enrichment analysis in Table 7.

**Table 6:** The Hub gene of the NF data subset.

**Module**	**Hub gene**	**Module**	**Hub gene**
Bisque4	Prr14	Lightyellow	Pml
Black	Tmem8b	Magenta	Acvr2a
Blue	BC052040	Mediumpurple3	Myo1b
Brown	Cntn2	Midnightblue	Atrx
Brown4	BC021891	Orange	Mybl1
Cyan	Tmem130	Orangered4	Acbd3
Darkgreen	Robo3	Paleturquoise	Taf1c
Darkgrey	Mboat1	Pink	Gm13375
Darkmagenta	Fam70b	Plum1	Pkn2
Darkolivegreen	Slc7a8	Plum2	Mmadhc
Darkorange	Slc25a47	Purple	Hhex
Darkorange2	Chd8	Red	Apc2
Darkred	Gpx2.ps1	Royalblue	Med26
Darkslateblue	Catsper2	Saddlebrown	Slc25a44
Darkturquoise	Ebf4	Salmon	Orm3
Floralwhite	Klhl28	Sienna3	Scube2
Green	Grem2	Skyblue	Nanp
Greenyellow	Usp18	Skyblue3	Nek11
Grey60	Zcchc12	Steelblue	Crx
Ivory	Ep300	Tan	Zfp651
Lightcyan	Ptgds	Turquoise	Ap3m2
Lightcyan1	mt.Rnr2	Violet	Qk
Lightgreen	Gstm2	White	B230217O12Rik
Lightsteelblue1	Disp1	Yellow	Inpp4a
		Yellowgreen	Zswim1

**Table 7:** Functional enrichment analysis of Hub genes in NF data subsets.

**Category**	**Term**	**Count**	***P*-value**	**Fold enrichment**	**FDR**
BP	GO:0045944	9	9.13E-04	4.19373792	0.233863747
BP	GO:0006351	12	0.001284966	2.951560906	0.233863747
BP	GO:0007275	8	0.005066806	3.604594952	0.422224333
BP	GO:0030154	7	0.005358539	4.160880999	0.422224333
BP	GO:0006355	12	0.005799785	2.441286664	0.422224333
BP	GO:0032206	2	0.020822877	92.72820513	1
BP	GO:0006810	9	0.032934936	2.290213628	1
BP	GO:0042771	2	0.063185914	29.91232423	1
BP	GO:0016055	3	0.07364446	6.530155291	1
CC	GO:0005634	18	0.059352202	1.469995016	1
CC	GO:0032993	2	0.059738529	31.71290323	1
CC	GO:0005654	8	0.083476947	2.032248062	1
MF	GO:0003677	10	0.010936964	2.55286147	0.677053596
MF	GO:0008013	3	0.012979407	16.83976834	0.677053596
MF	GO:0003682	5	0.015065842	5.05915787	0.677053596
MF	GO:0032183	2	0.018423227	104.7807808	0.677053596
MF	GO:0000978	4	0.037404986	5.253632463	1
MF	GO:0035257	2	0.048397769	39.29279279	1
MF	GO:0003713	3	0.053050255	7.858558559	1
MF	GO:0004674	4	0.057674865	4.406668351	1
MF	GO:0000977	3	0.07599868	6.40063593	1
MF	GO:0016740	7	0.078508897	2.242251763	1
MF	GO:0005524	7	0.085817386	2.190175577	1
MF	GO:0035064	2	0.092637771	20.06440483	1
MF	GO:0004672	4	0.09568683	3.551890874	1

### Differential Network Analysis With DiffCoEx

**Figure 4:**
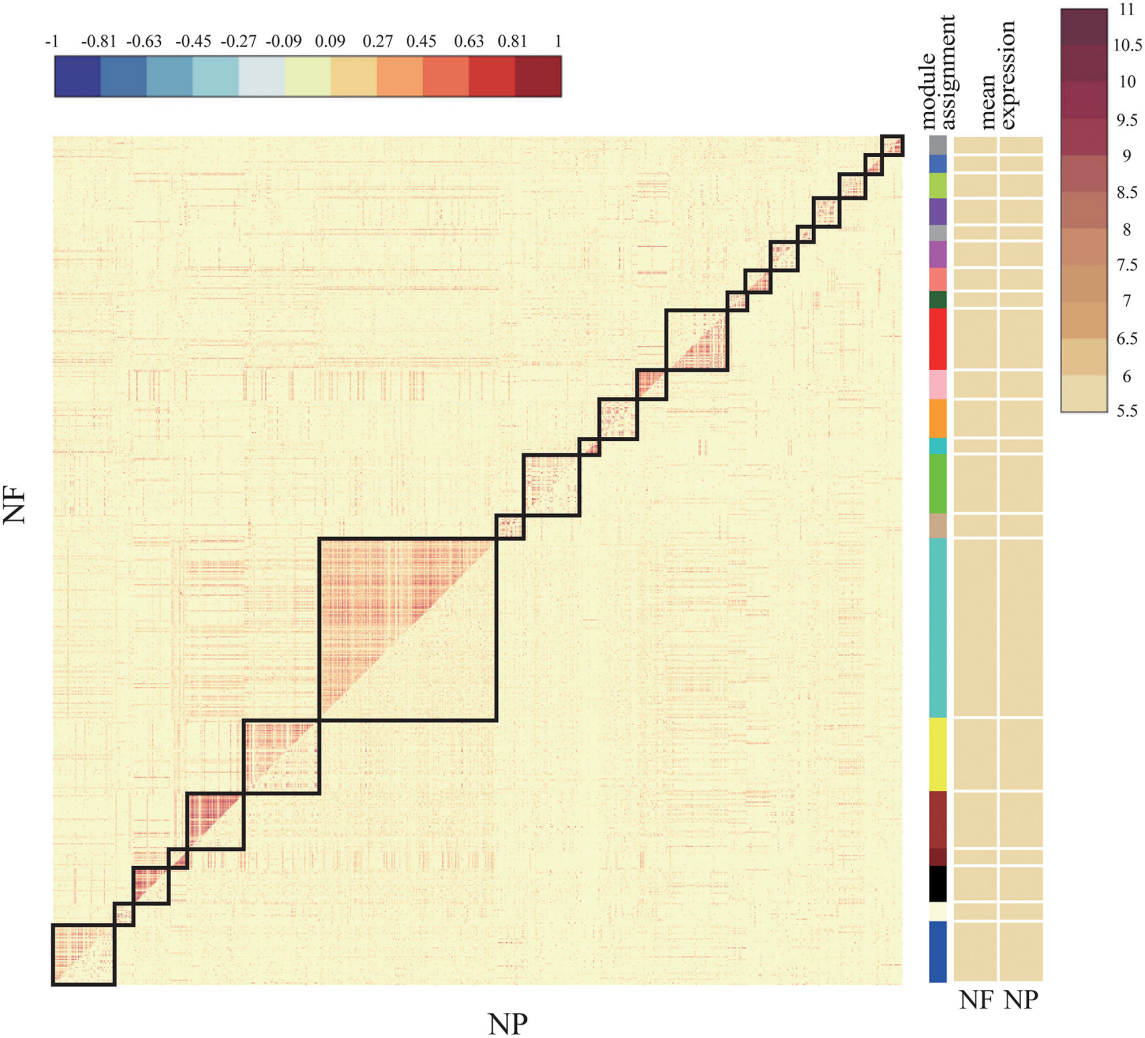
Comparative correlation heat map of NF and NP. The upper diagonal of the main matrix shows a correlation between pairs of genes among the NF (the red color corresponds to positive correlations, blue to negative correlations). The lower diagonal of the heat map shows a correlation between the same gene pairs in the NP controls. Modules are identified in the heat map by black squares and on the right side of the heat map by a color bar. The brown bands on the right side indicate the mean expression of the modules in the NF (first column) and the NP (second column); darker colors indicate higher mean expression levels.

When we use DiffCoEx to analyze the difference network of each two types of Usoskin and Xin data, using the default parameters will lead to too many modules. In order to reduce the number of modules as much as possible, the gene is sampled, in other words, only 1/2 of the genes were randomly selected as the input to DiffCoEx, and the “cutHeight” parameter of the “mergeCloseModules” function was adjusted to 0.5 (default 0.2). Here, we only show the results of the two data types of NF-NP in the Usoskin dataset, as shown in Figure 4. The Xin data set and the other five analysis results are show in Supplementary Material 3.

The upper half of the main matrix in the figure shows the relationship between genes and genes in the NF subset, and the lower half shows the relationship between genes and genes in the NP subset. There are a total of 21 modules in the figure. Some modules have a higher expression level in NF and some have higher expression levels in NP. The color difference between the two sides is more obvious, indicating that this module has a large difference between NF and NP, which can be targeted at the difference. Analysis of the more obvious modules plays a vital role in the exploration of downstream target genes and drug prediction.

## Discussion

In recent years, the rapid development of single-cell sequencing technology can simultaneously measure the expression levels of tens of thousands of cells in a single experiment. Because of this, single-cell sequencing technology has developed rapidly in recent years. Although a large number of research methods have been developed for single-cell sequencing technology, there is no systematic framework on how to compare two single-cell clusters at the molecular level. Due to the difference in gene expression levels, different cells have different biological meanings and different physiological functions. Each gene is involved in a different biological process. It is not feasible to analyze all genes blindly to predict drugs and treat diseases. Therefore, analyzing data from the perspective of genetics plays an important role in clinical trials and scientific research. In this work, we performed a complete process analysis of scRNA-seq data at the molecular level. For example, through DEGs, we can know whether there are differences between different groups, and which genes are different. Furthermore, the functional enrichment analysis (GO, KEGG) of these differential genes was performed to explore the relevant signal pathways and the biological processes mediated by the differences in the expression of these genes. By constructing a gene regulatory network (WGCNA), it is helpful to understand the function of different genes and the interaction between genes as a whole, to better understand the gene expression mechanism inside cells, and to promote the research of disease pathology. By analyzing the difference modules in the entire gene regulatory network, exploring modules that contain more biological information provides effective guidance for the prediction of targeted genes and subsequent analysis.

This work mainly focuses on the analysis of the gene level in single-cell data, including the analysis of differential genes, the analysis of differential correlation, the construction of gene regulatory networks and the analysis of differential networks, without considering the internal dynamics between cells. How to effectively express the biological information contained in genes and cells in words is one of our future research directions. And due to the lack of relevant biological background knowledge, the analysis and description of the analysis results and the regulatory relationship between genes are insufficient. At the same time, more algorithm models can be considered for constructing the relationship between genes.

## Data Availability Statement

The datasets presented in this study can be found in online repositories. The names of the repository/repositories and accession number(s) can be found in the article/Supplementary Material.

## Author Contributions

WL, HA, and LC conceived, designed, and managed the study. WL and LC performed the experiments and drafted the manuscript. BW, CR, and AW provided computational support and technical assistance. All authors reviewed and approved the final manuscript.

## Conflict of Interest

BW and AW were employed by the company Geneis Beijing Co., Ltd. HA was employed by the company Guangzhou Anjie Biomedical Technology Co., Ltd. The remaining authors declare that the research was conducted in the absence of any commercial or financial relationships that could be construed as a potential conflict of interest.
