# Supplementary material for: A Novel Method to Identify the Differences Between Two Single Cell Groups at Single Gene, Gene Pair, and Gene Module Levels
Source: Front Genet. 2021 Mar 15;12:648898. doi: 10.3389/fgene.2021.648898 (PMC8005607; doi:10.3389/fgene.2021.648898)
Supplement: Supplementary file 1 [file Data_Sheet_1.PDF]

Supplementary Material

1 GO and KEGG analysis were performed on the differential genes

In this work, we use DEsingle and SigEMD two methods to analyze the four types of data contained in Usoskin and Xin, overlap the differential genes obtained by the two methods. In addition, we performed two enrichment analyses of GO and KEGG on the top 20 differential genes with overlapping data in each two groups. We show here the enrichment analysis results of NF-PEP, NF-TH, NP-PEP, NP-TH, PEP-TH and the result of Xin dataset.

(1)NF-PEP:

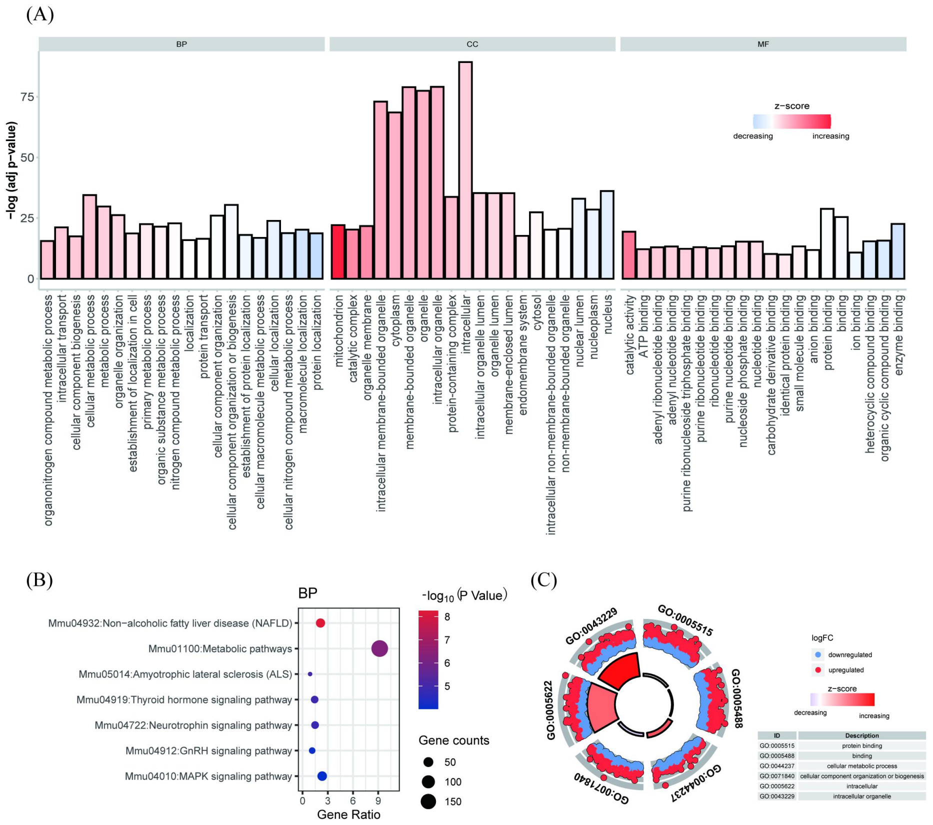

Supplementary Figure 1. (A) Perform enrichment analysis on the differential genes overlapping NF-PEP data, and display the top 20 most significant terms in BP, CC, and MF. **(B)** Perform KEGG enrichment analysis on the differential genes with overlapping NF-PEP data. **(C)** Basic information of 6 specified terms, among them, blue means down-regulated genes, red means up-regulated genes.

(2) NF-TH:

(A)

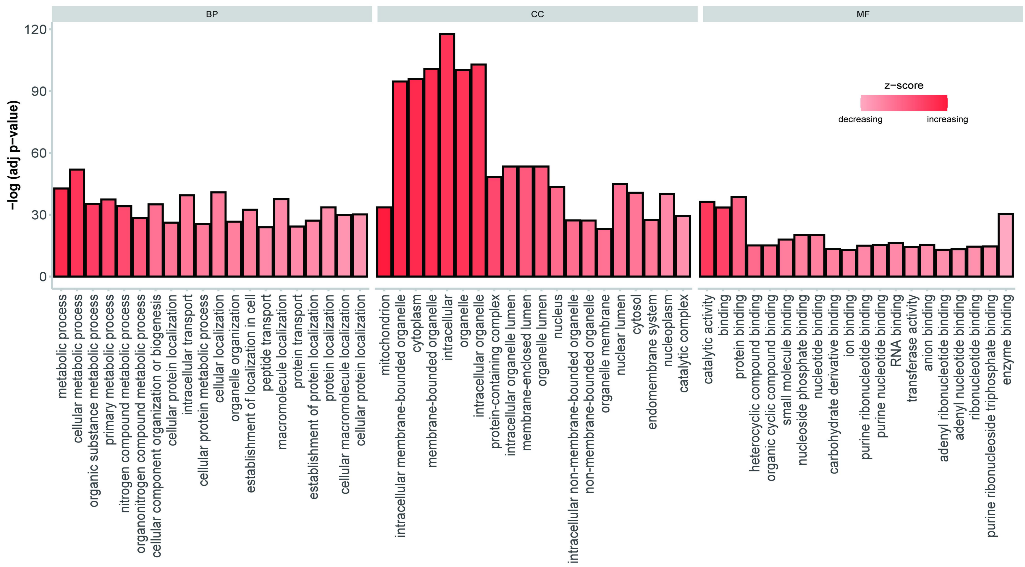

(B)

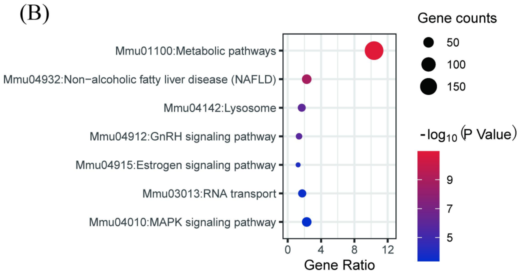

(C)

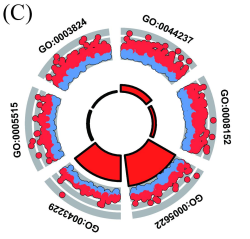

ID	Description
GO:0044237	cellular metabolic process
GO:0008152	metabolic process
GO:0005622	intracellular
GO:0043229	intracellular organelle
GO:0005515	protein binding
GO:0003824	catalytic activity

Supplementary Figure 2. (A) Perform enrichment analysis on the differential genes overlapping NF-TH data, and display the top 20 most significant terms in BP, CC, and MF. **(B)** Perform KEGG enrichment analysis on the differential genes with overlapping NF-TH data. **(C)** Basic information of 6 specified terms, among them, blue means down-regulated genes, red means up-regulated genes.

(3)NP-PEP:

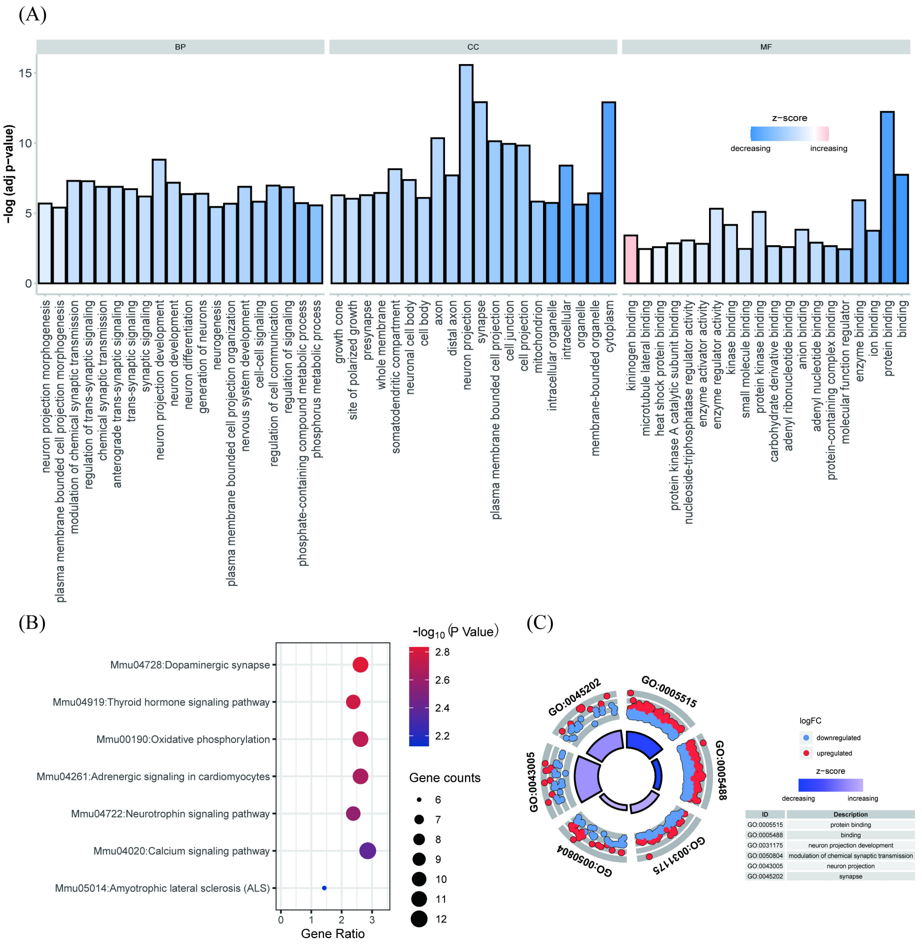

Supplementary Figure3. (A) Perform enrichment analysis on the differential genes overlapping NP-PEP data, and display the top 20 most significant terms in BP, CC, and MF. **(B)** Perform KEGG enrichment analysis on the differential genes with overlapping NP-PEP data. **(C)** Basic information of 6 specified terms, among them, blue means down-regulated genes, red means up-regulated genes.

(4)NP-TH:

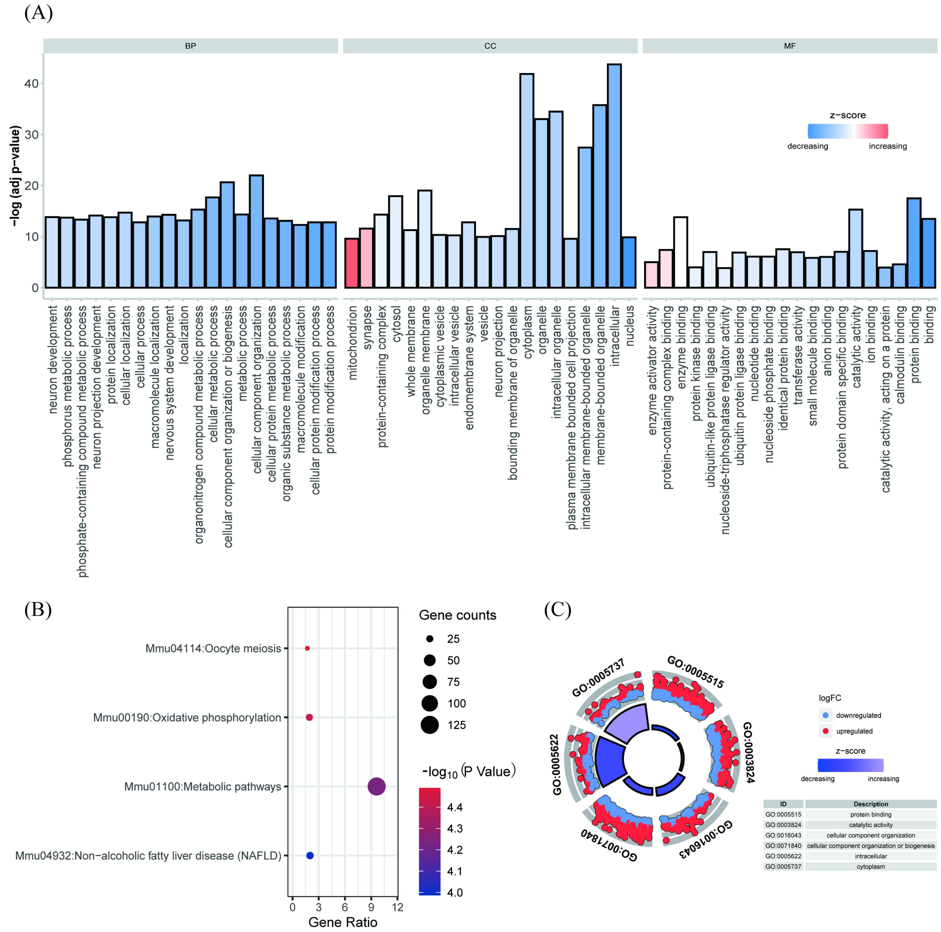

Supplementary Figure 4. (A) Perform enrichment analysis on the differential genes overlapping NP-TH data, and display the top 20 most significant terms in BP, CC, and MF. **(B)** Perform KEGG enrichment analysis on the differential genes with overlapping NP-TH data. **(C)** Basic information of 6 specified terms, among them, blue means down-regulated genes, red means up-regulated genes.

(5)PEP-TH:

(A)

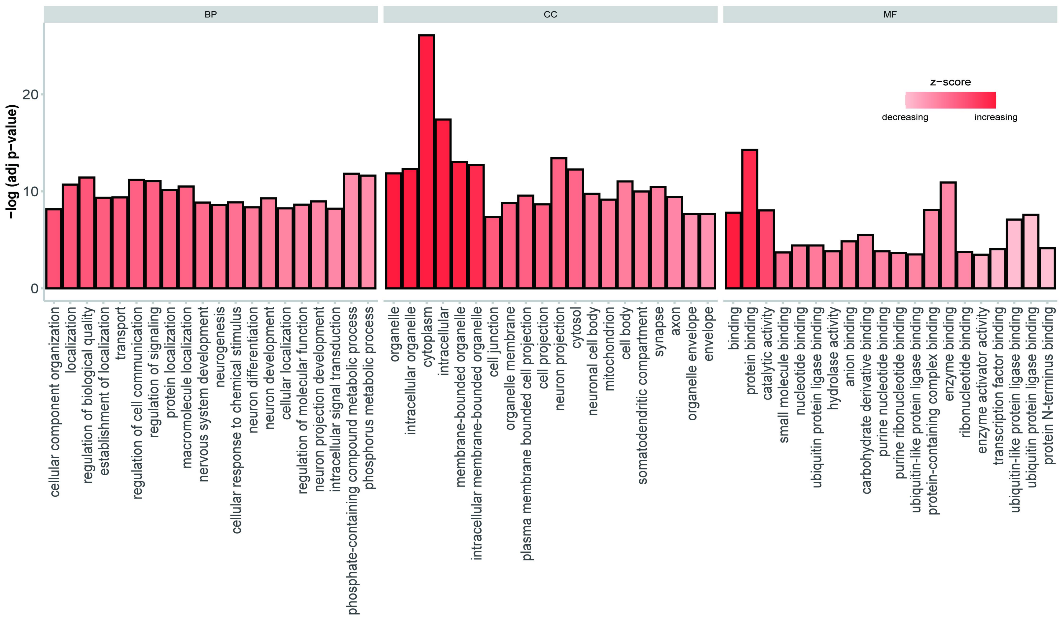

(B)

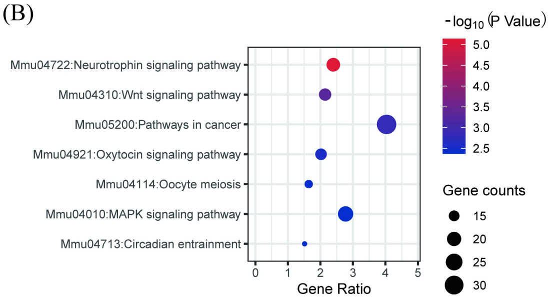

(C)

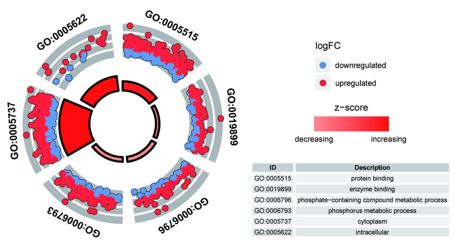

Supplementary Figure 5. (A) Perform enrichment analysis on the differential genes overlapping PEP-TH data, and display the top 20 most significant terms in BP, CC, and MF. **(B)** Perform KEGG enrichment analysis on the differential genes with overlapping PEP-TH data. **(C)** Basic information of 6 specified terms, among them, blue means down-regulated genes, red means up-regulated genes.

(6)alpha-beta:

(A)

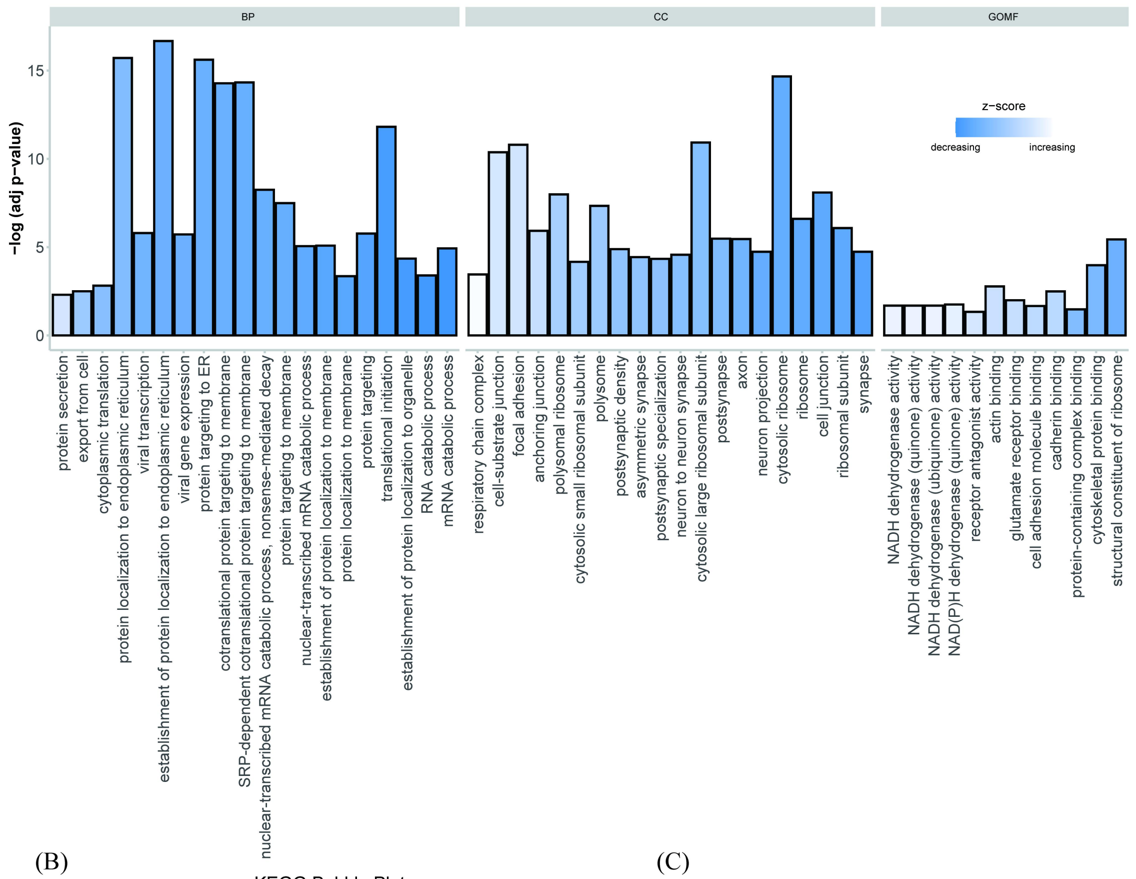

(B)

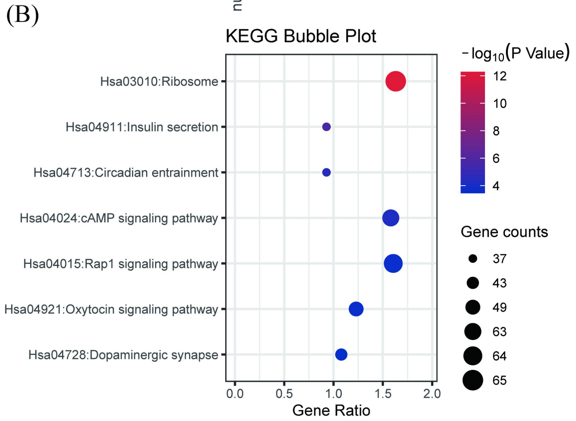

(C)

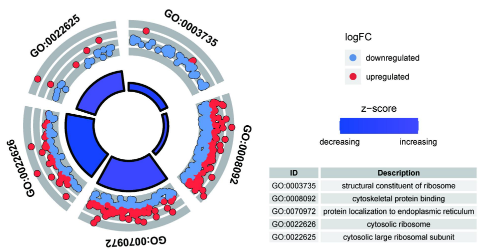

Supplementary Figure 6. (A) Perform enrichment analysis on the differential genes overlapping alpha-beta data, and display the top 20 most significant terms in BP, CC, and MF. **(B)** Perform KEGG enrichment analysis on the differential genes with overlapping alpha-beta data. **(C)** Basic information of 6 specified terms, among them, blue means down-regulated genes, red means up-regulated genes.

(7)alpha-delta:

(A)

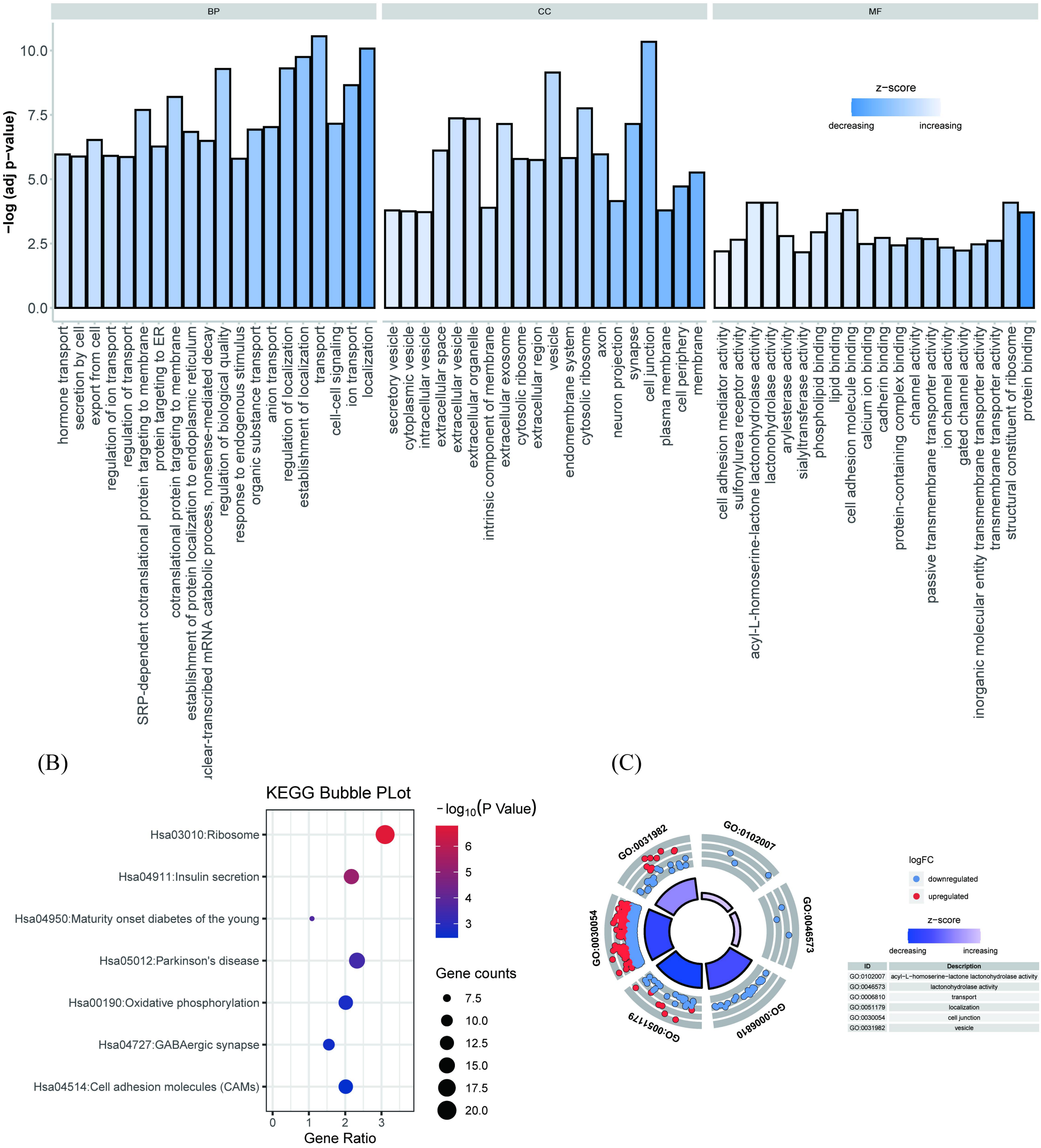

Supplementary Figure 7. (A) Perform enrichment analysis on the differential genes overlapping alpha-delta data, and display the top 20 most significant terms in BP, CC, and MF. **(B)** Perform KEGG enrichment analysis on the differential genes with overlapping alpha-delta data. **(C)** Basic information of 6 specified terms, among them, blue means down-regulated genes, red means up-regulated genes.

(8)alpha-pp:

(A)

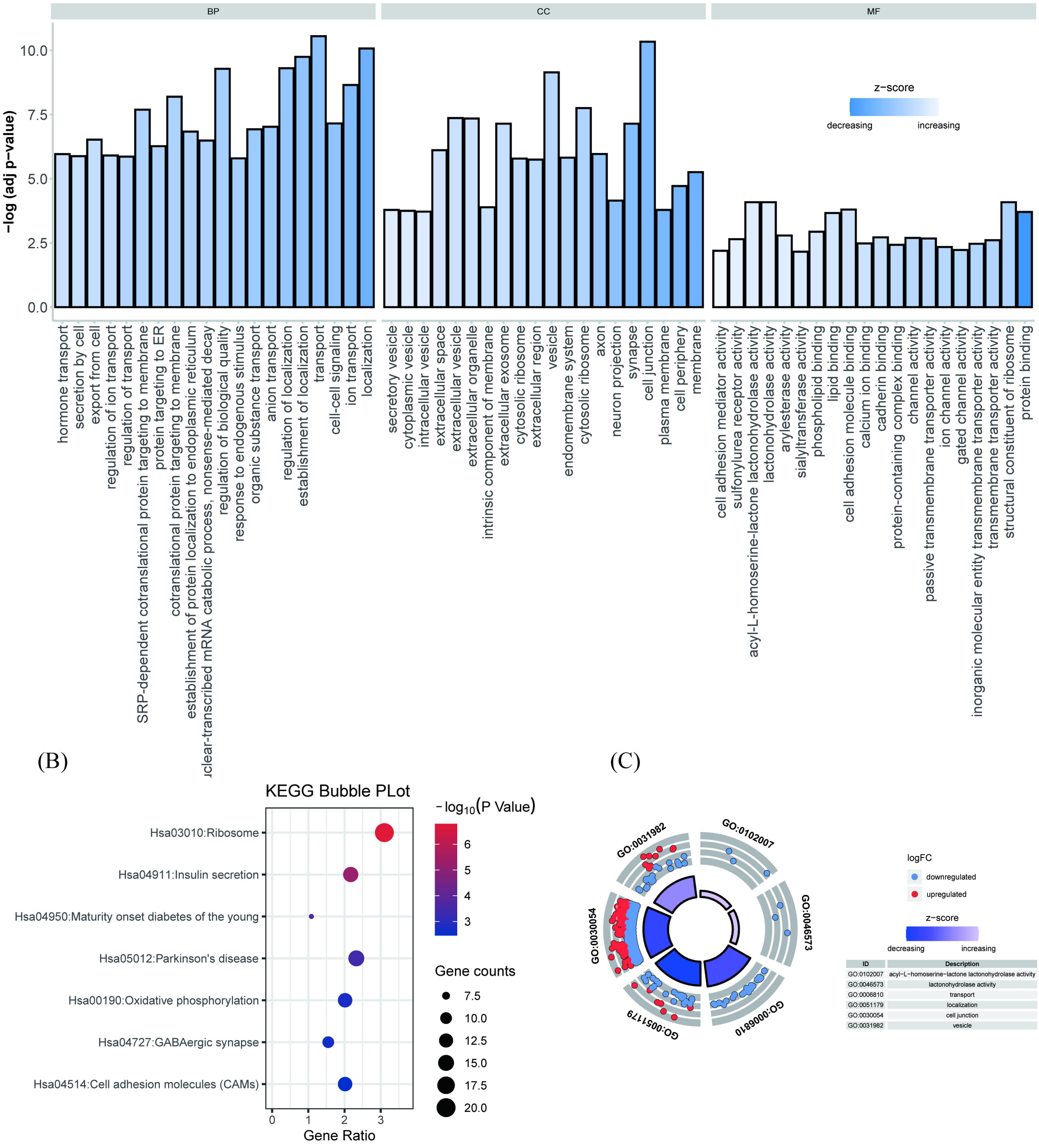

(B)

(C)

Supplementary Figure 8. (A) Perform enrichment analysis on the differential genes overlapping alpha-pp data, and display the top 20 most significant terms in BP, CC, and MF. **(B)** Perform KEGG enrichment analysis on the differential genes with overlapping alpha-pp data. **(C)** Basic information of 6 specified terms, among them, blue means down-regulated genes, red means up-regulated genes.

(9)beta-delta:

(A)

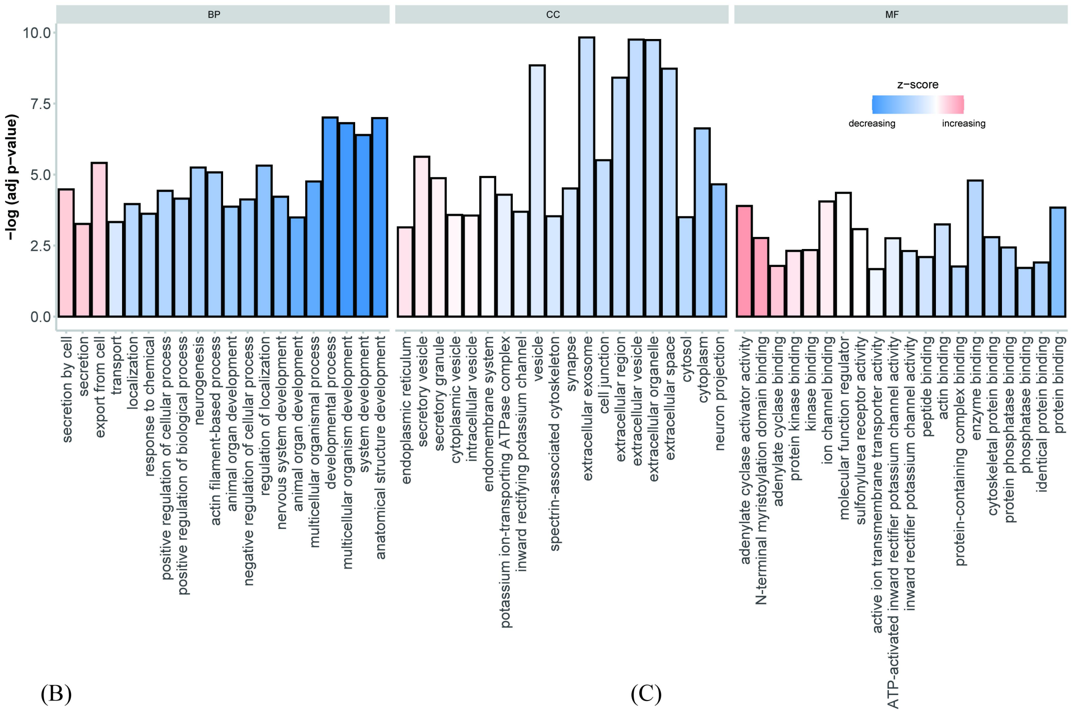

(B)

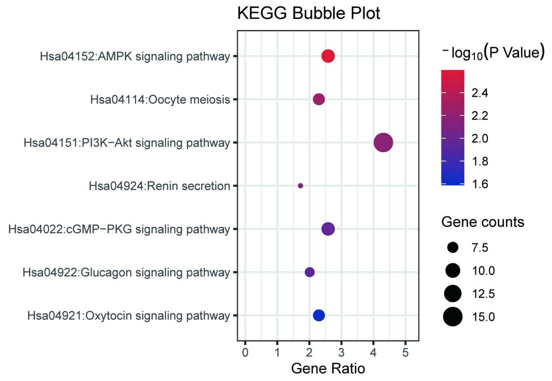

(C)

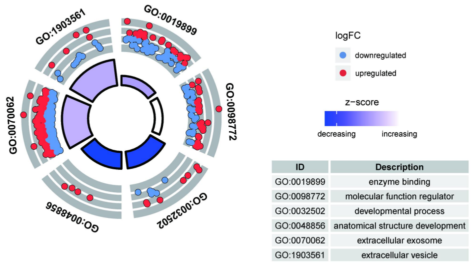

Supplementary Figure 9. (A) Perform enrichment analysis on the differential genes overlapping beta-delta data, and display the top 20 most significant terms in BP, CC, and MF. **(B)** Perform KEGG enrichment analysis on the differential genes with overlapping beta-delta data. **(C)** Basic information of 6 specified terms, among them, blue means down-regulated genes, red means up-regulated genes.

(10)beta-pp:

(A)

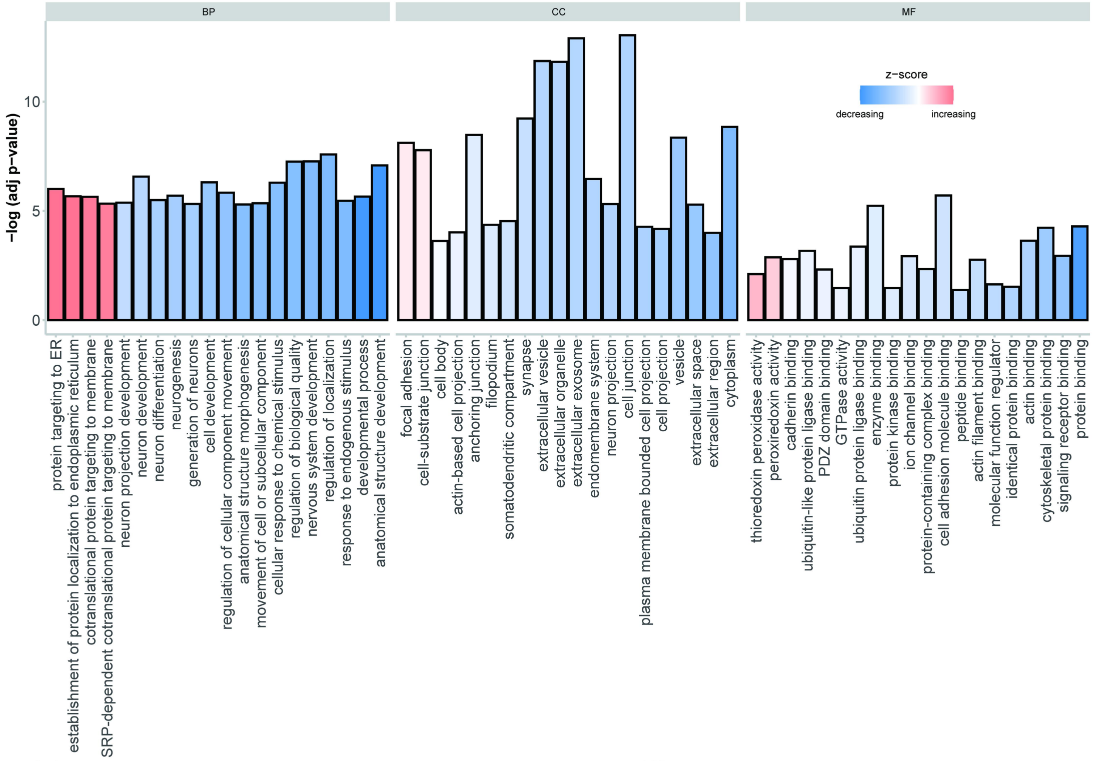

(B)

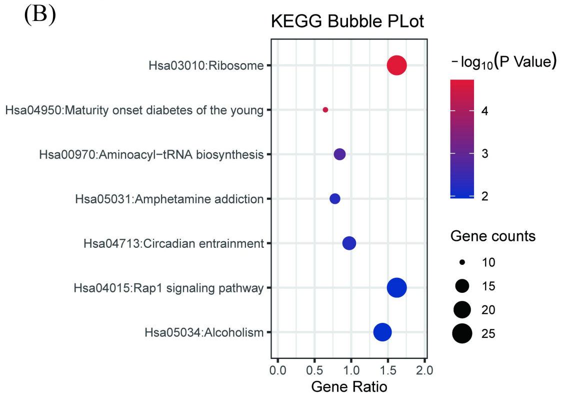

(C)

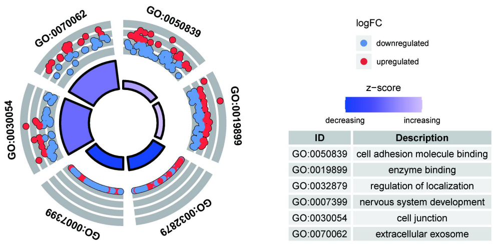

Supplementary Figure 10. (A) Perform enrichment analysis on the differential genes overlapping beta-pp data, and display the top 20 most significant terms in BP, CC, and MF. **(B)** Perform KEGG enrichment analysis on the differential genes with overlapping beta-pp data. **(C)** Basic information of 6 specified terms, among them, blue means down-regulated genes, red means up-regulated genes.

(11)delta-pp:

(A)

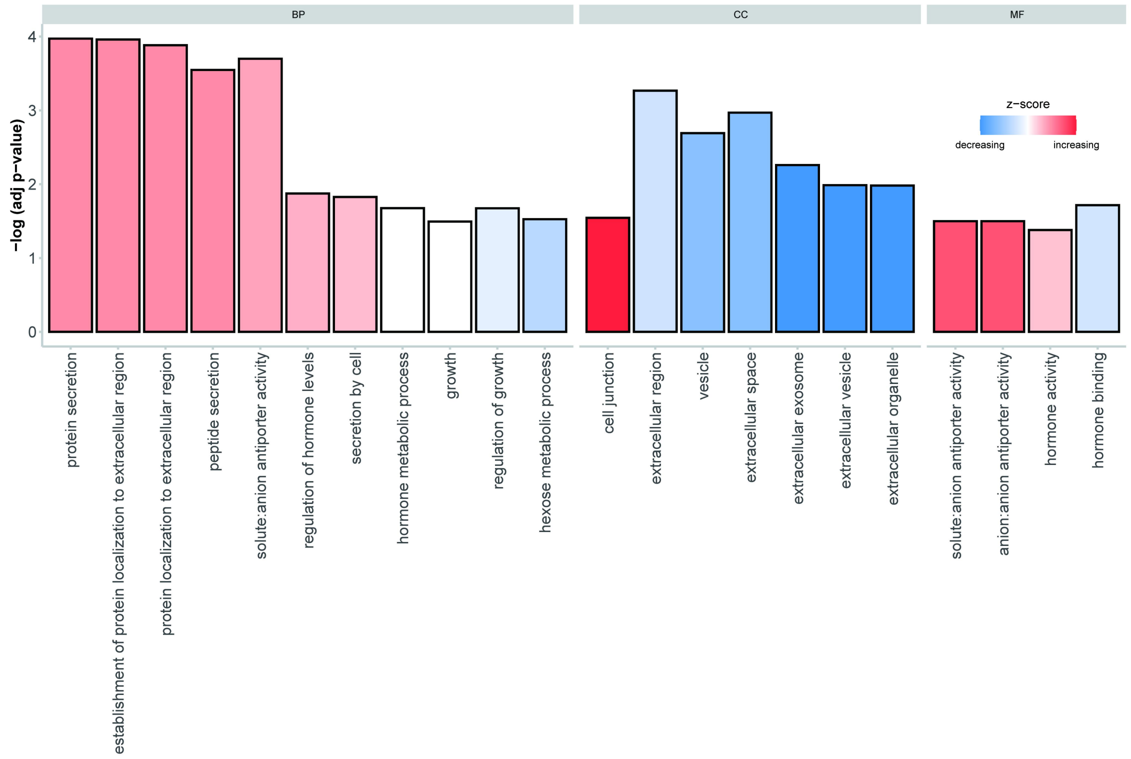

(B)

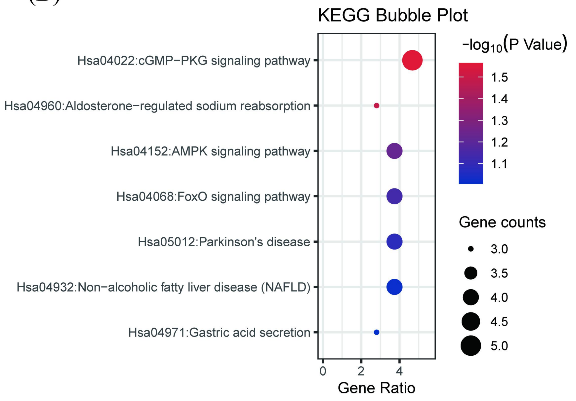

(C)

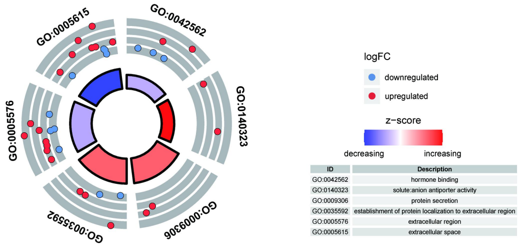

Supplementary Figure 11. (A) Perform enrichment analysis on the differential genes overlapping delta-pp data, and display the top 20 most significant terms in BP, CC, and MF. **(B)** Perform KEGG enrichment analysis on the differential genes with overlapping delta-pp data. **(C)** Basic information of 6 specified terms, among them, blue means down-regulated genes, red means up-regulated genes.

2 Network analysis

(1)NP-TH:

(A) (B)

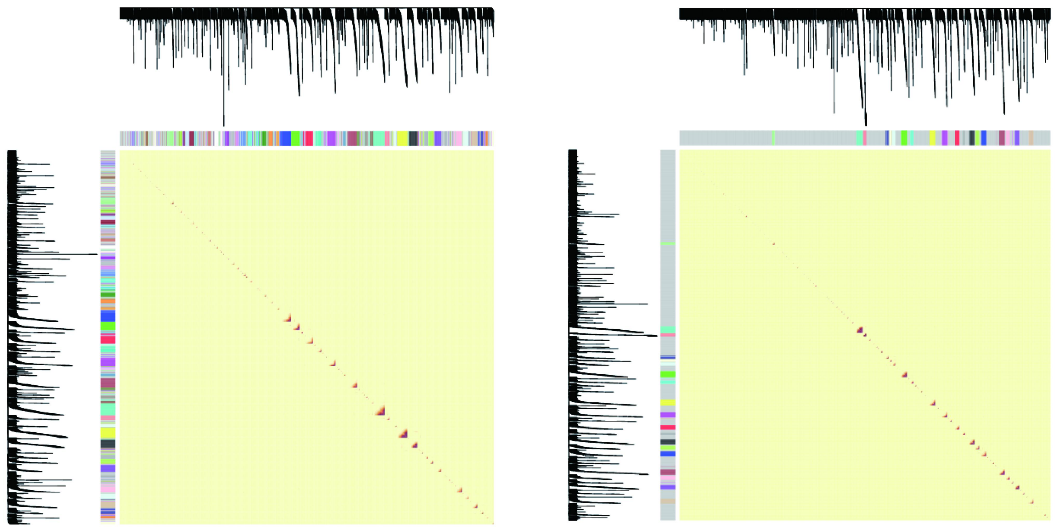

Supplementary Figure 12. (A) and **(B)** are network heatmaps of NP and TH respectively. On the left side and top are the hierarchical clustering trees and modules of genes. In the figure, red represents higher similarity and yellow represents lower similarity. As the module is composed of genes with high similarity, it corresponds to the diagonal red in the figure.

(2)Xin:

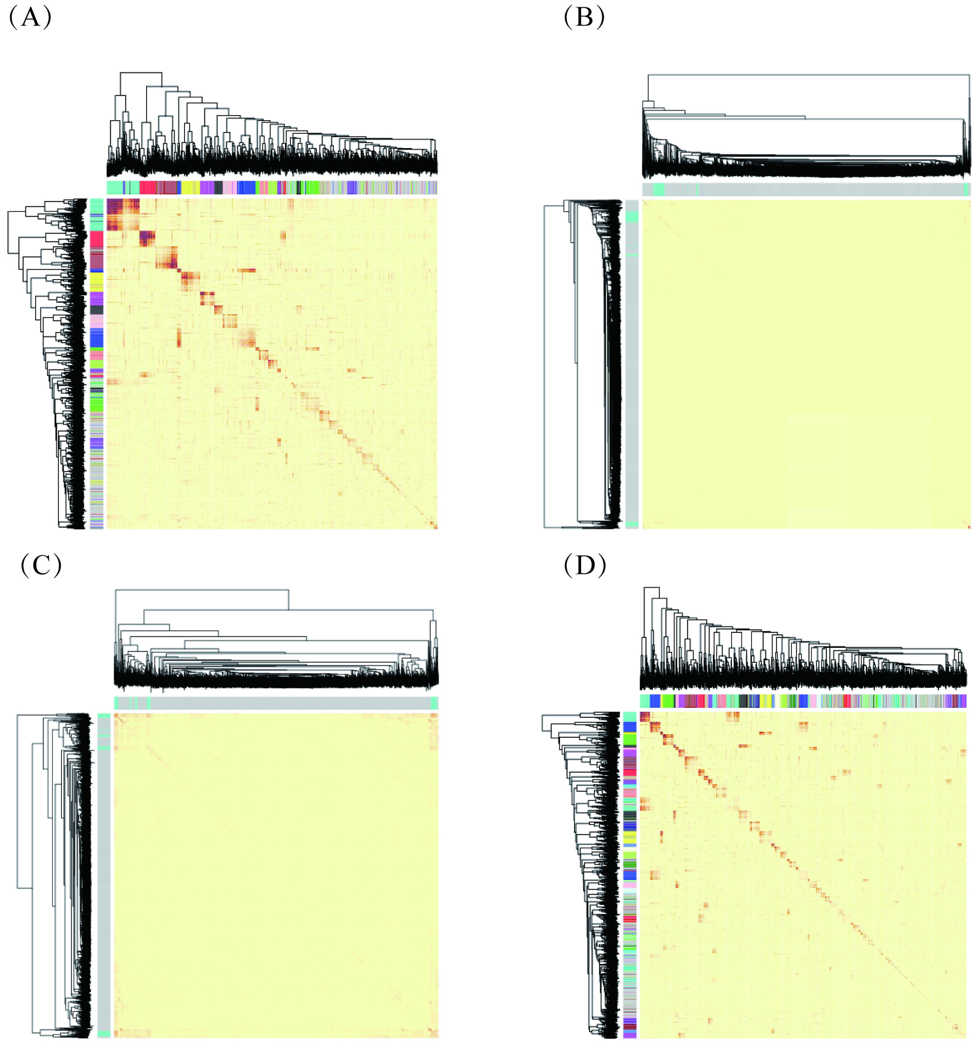

Supplementary Figure 13. (A), (B), (C) and **(D)** are network heatmaps of delta, alpha, beta and pp respectively. On the left side and top are the hierarchical clustering trees and modules of genes. In the figure, red represents higher similarity and yellow represents lower similarity. As the module is composed of genes with high similarity, it corresponds to the diagonal red in the figure.

3 Differential network analysis

(1)NF-PEP:

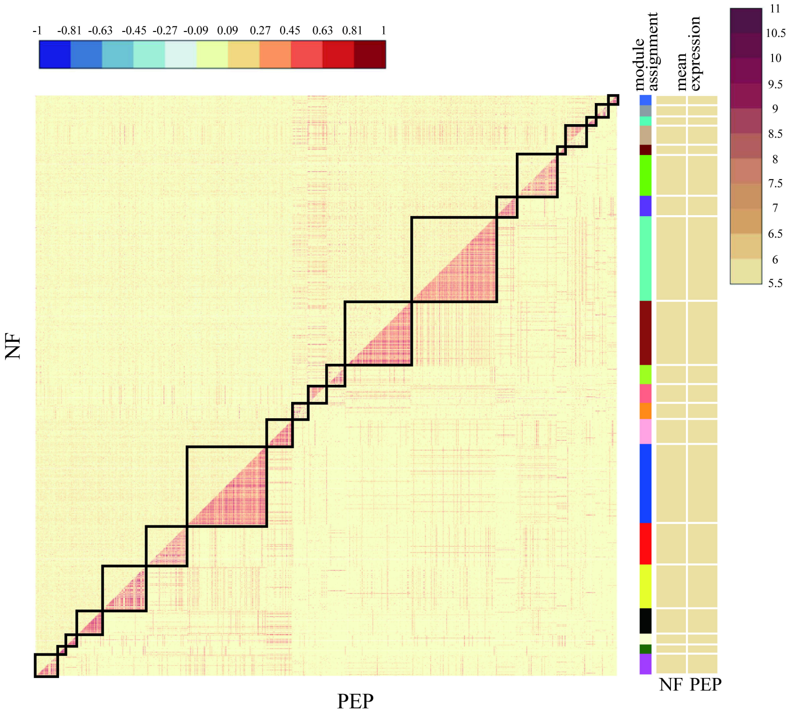

Supplementary Figure 14. Comparative correlation heat map of NF and PEP. The upper diagonal of the main matrix shows a correlation between pairs of genes among the NF (the red color corresponds to positive correlations, blue to negative correlations). The lower diagonal of the heat map shows a correlation between the same gene pairs in the PEP controls. Modules are identified in the heat map by black squares and on the right side of the heat map by a color bar. The brown bands on the right side indicate the mean expression of the modules in the NF (first column) and the PEP(second column); darker colors indicate higher mean expression levels.

(2)NF-TH:

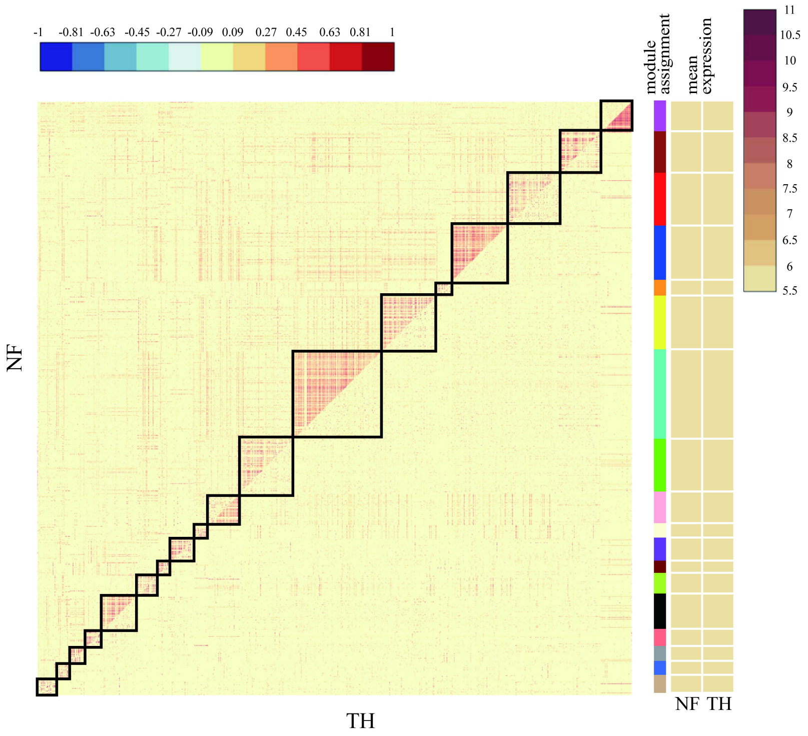

Supplementary Figure 15. Comparative correlation heat map of NF and TH. The upper diagonal of the main matrix shows a correlation between pairs of genes among the NF (the red color corresponds to positive correlations, blue to negative correlations). The lower diagonal of the heat map shows a correlation between the same gene pairs in the TH controls. Modules are identified in the heat map by black squares and on the right side of the heat map by a color bar. The brown bands on the right side indicate the mean expression of the modules in the NF (first column) and the TH (second column); darker colors indicate higher mean expression levels.

(3)NP-PEP:

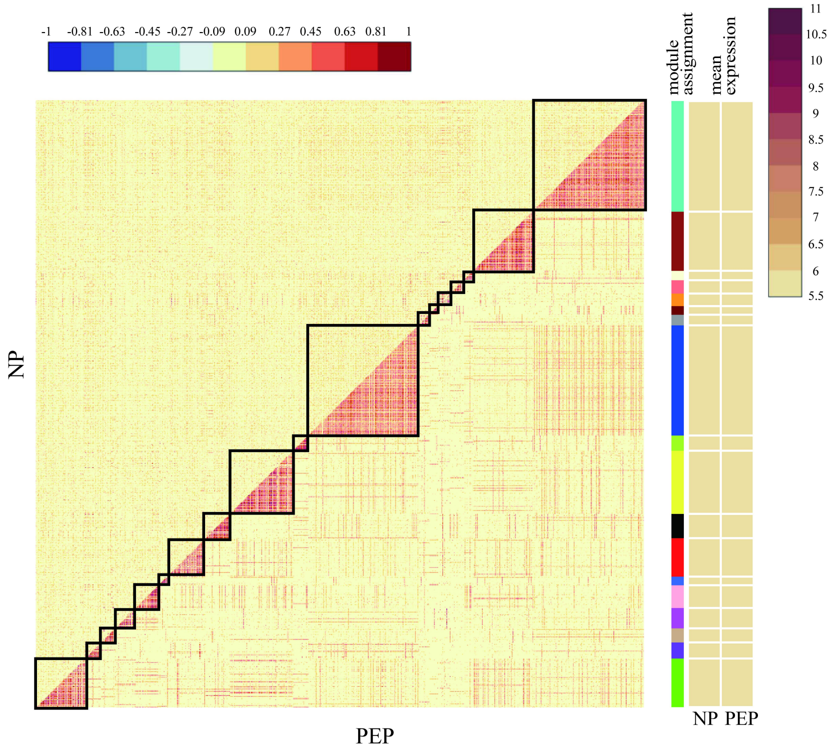

Supplementary Figure 16. Comparative correlation heat map of NP and PEP. The upper diagonal of the main matrix shows a correlation between pairs of genes among the NP (the red color corresponds to positive correlations, blue to negative correlations). The lower diagonal of the heat map shows a correlation between the same gene pairs in the PEP controls. Modules are identified in the heat map by black squares and on the right side of the heat map by a color bar. The brown bands on the right side indicate the mean expression of the modules in the NP (first column) and the PEP (second column); darker colors indicate higher mean expression levels.

(4)NP-TH:

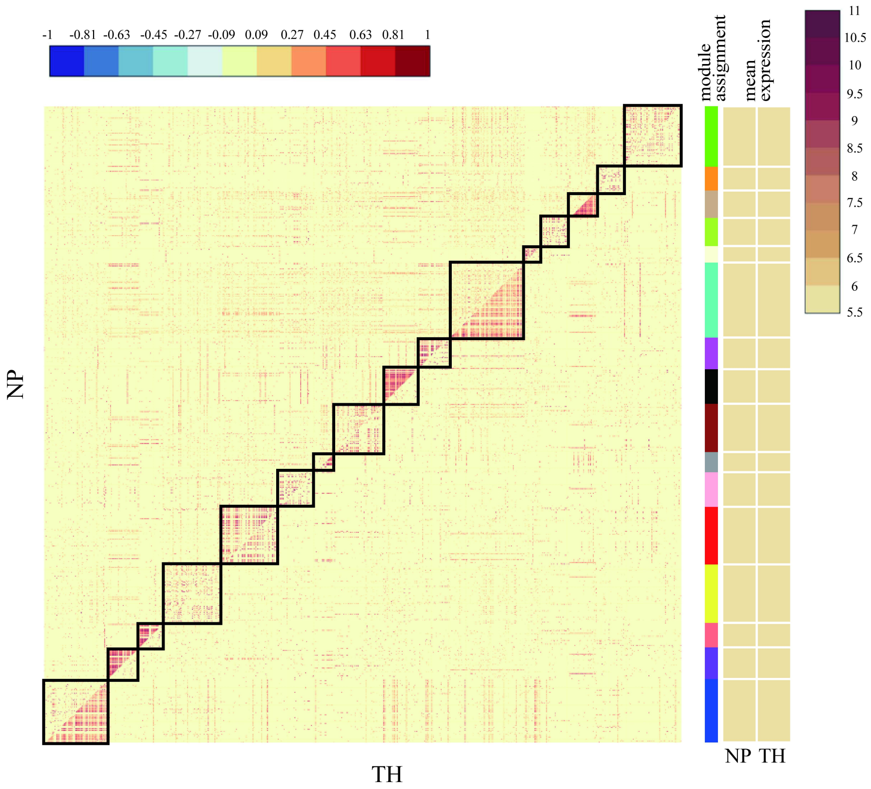

Supplementary Figure 17. Comparative correlation heat map of NP and TH. The upper diagonal of the main matrix shows a correlation between pairs of genes among the NP (the red color corresponds to positive correlations, blue to negative correlations). The lower diagonal of the heat map shows a correlation between the same gene pairs in the TH controls. Modules are identified in the heat map by black squares and on the right side of the heat map by a color bar. The brown bands on the right side indicate the mean expression of the modules in the NP (first column) and the TH (second column); darker colors indicate higher mean expression levels.

(5)PEP-TH:

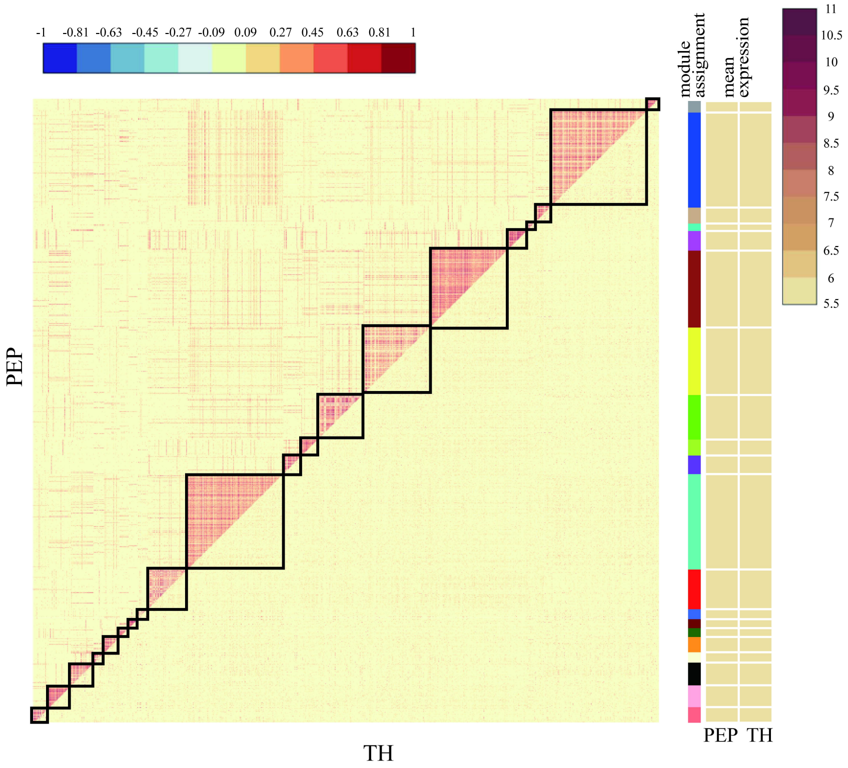

Supplementary Figure 18. Comparative correlation heat map of PEP and TH. The upper diagonal of the main matrix shows a correlation between pairs of genes among the PEP (the red color corresponds to positive correlations, blue to negative correlations). The lower diagonal of the heat map shows a correlation between the same gene pairs in the TH controls. Modules are identified in the heat map by black squares and on the right side of the heat map by a color bar. The brown bands on the right side indicate the mean expression of the modules in the PEP (first column) and the TH (second column); darker colors indicate higher mean expression levels.

(6)alpha-beta:

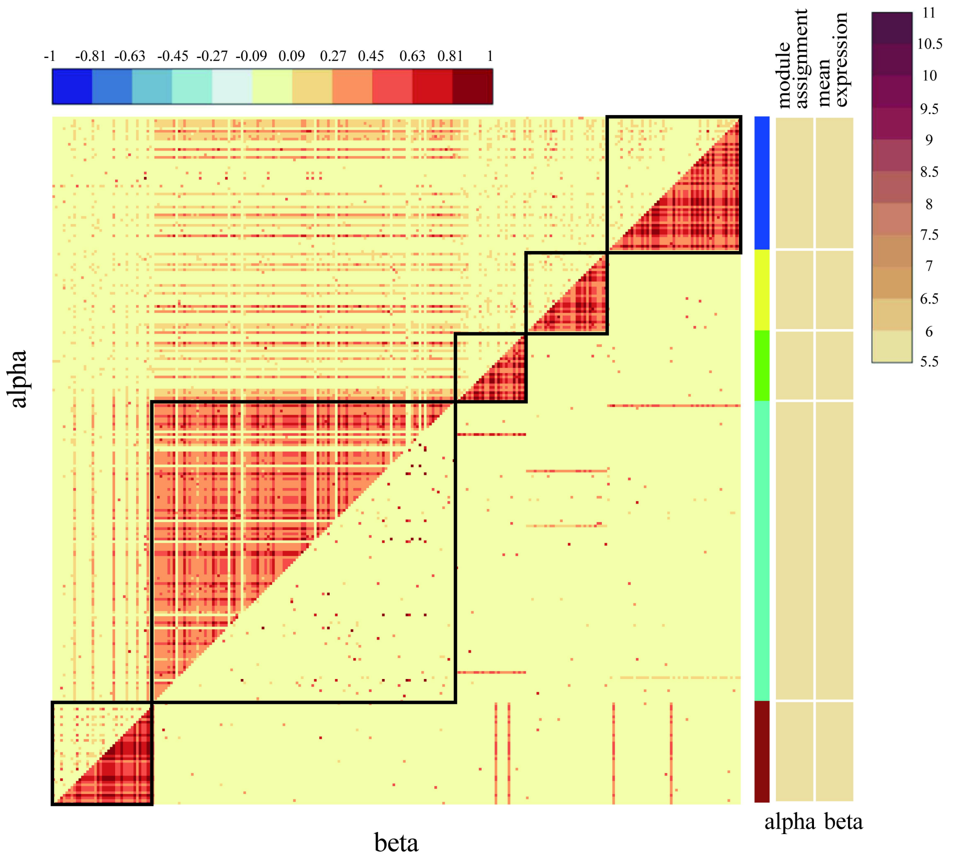

Supplementary Figure 19. Comparative correlation heat map of alpha and beta. The upper diagonal of the main matrix shows a correlation between pairs of genes among the alpha (the red color corresponds to positive correlations, blue to negative correlations). The lower diagonal of the heat map shows a correlation between the same gene pairs in the beta controls. Modules are identified in the heat map by black squares and on the right side of the heat map by a color bar. The brown bands on the right side indicate the mean expression of the modules in the alpha (first column) and the beta (second column); darker colors indicate higher mean expression levels.

(7)alpha-delta:

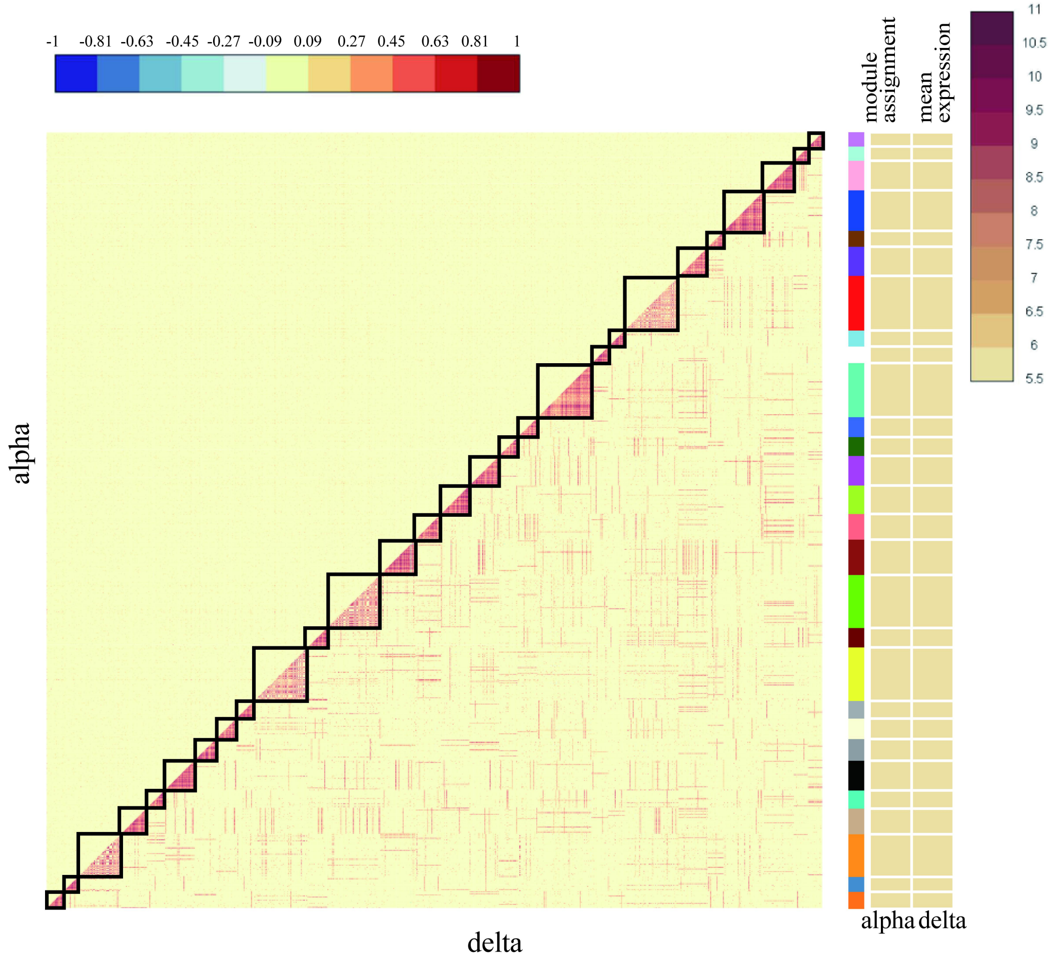

Supplementary Figure 20. Comparative correlation heat map of alpha and delta. The upper diagonal of the main matrix shows a correlation between pairs of genes among the alpha (the red color corresponds to positive correlations, blue to negative correlations). The lower diagonal of the heat map shows a correlation between the same gene pairs in the delta controls. Modules are identified in the heat map by black squares and on the right side of the heat map by a color bar. The brown bands on the right side indicate the mean expression of the modules in the alpha (first column) and the delta (second column); darker colors indicate higher mean expression levels.

(8)alpha-pp:

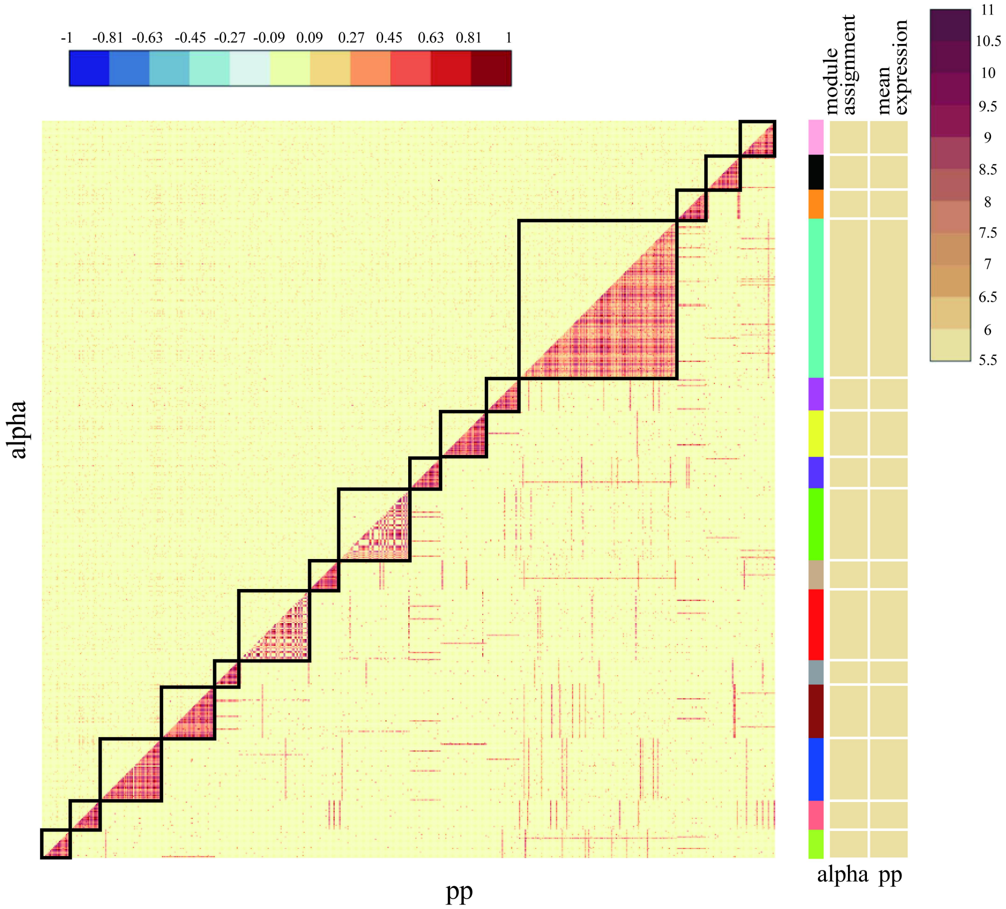

Supplementary Figure 21. Comparative correlation heat map of alpha and pp. The upper diagonal of the main matrix shows a correlation between pairs of genes among the alpha (the red color corresponds to positive correlations, blue to negative correlations). The lower diagonal of the heat map shows a correlation between the same gene pairs in the pp controls. Modules are identified in the heat map by black squares and on the right side of the heat map by a color bar. The brown bands on the right side indicate the mean expression of the modules in the alpha (first column) and the pp (second column); darker colors indicate higher mean expression levels.

(9)beta-delta:

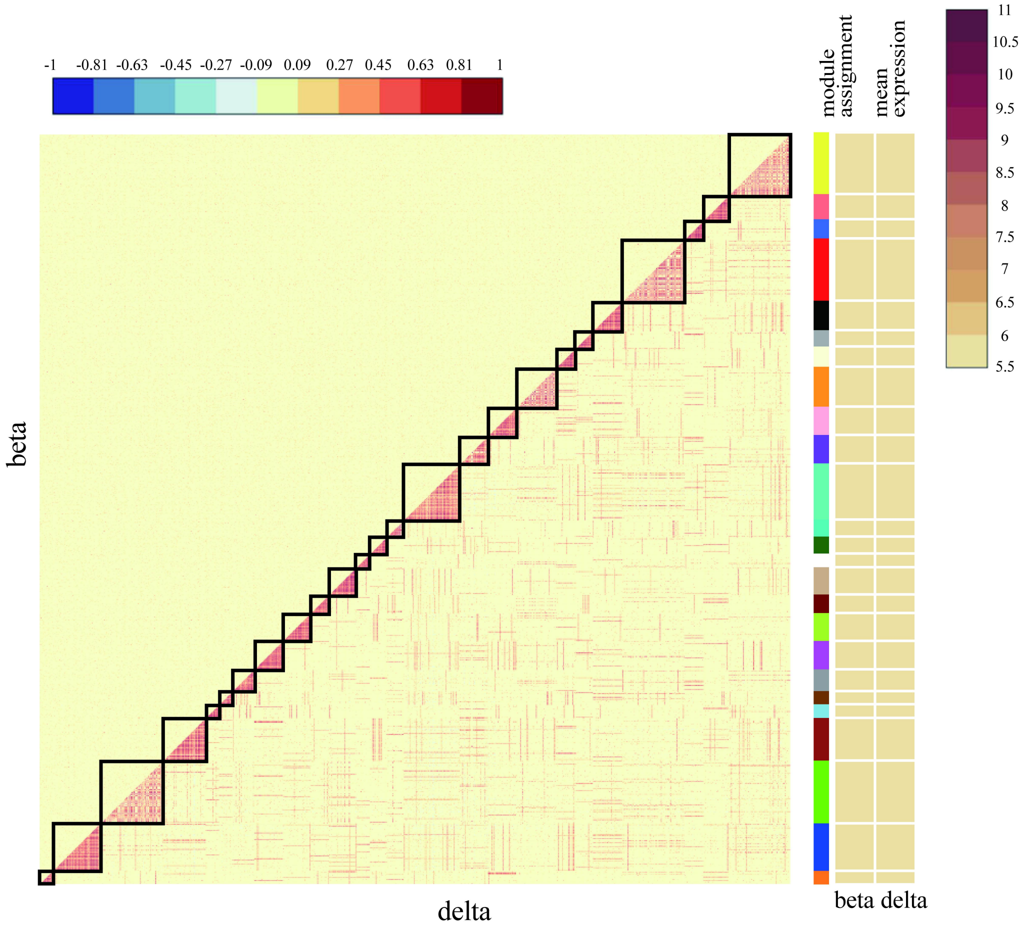

Supplementary Figure 22. Comparative correlation heat map of beta and delta. The upper diagonal of the main matrix shows a correlation between pairs of genes among the beta (the red color corresponds to positive correlations, blue to negative correlations). The lower diagonal of the heat map shows a correlation between the same gene pairs in the delta controls. Modules are identified in the heat map by black squares and on the right side of the heat map by a color bar. The brown bands on the right side indicate the mean expression of the modules in the beta (first column) and the delta (second column); darker colors indicate higher mean expression levels.

(10)beta-pp:

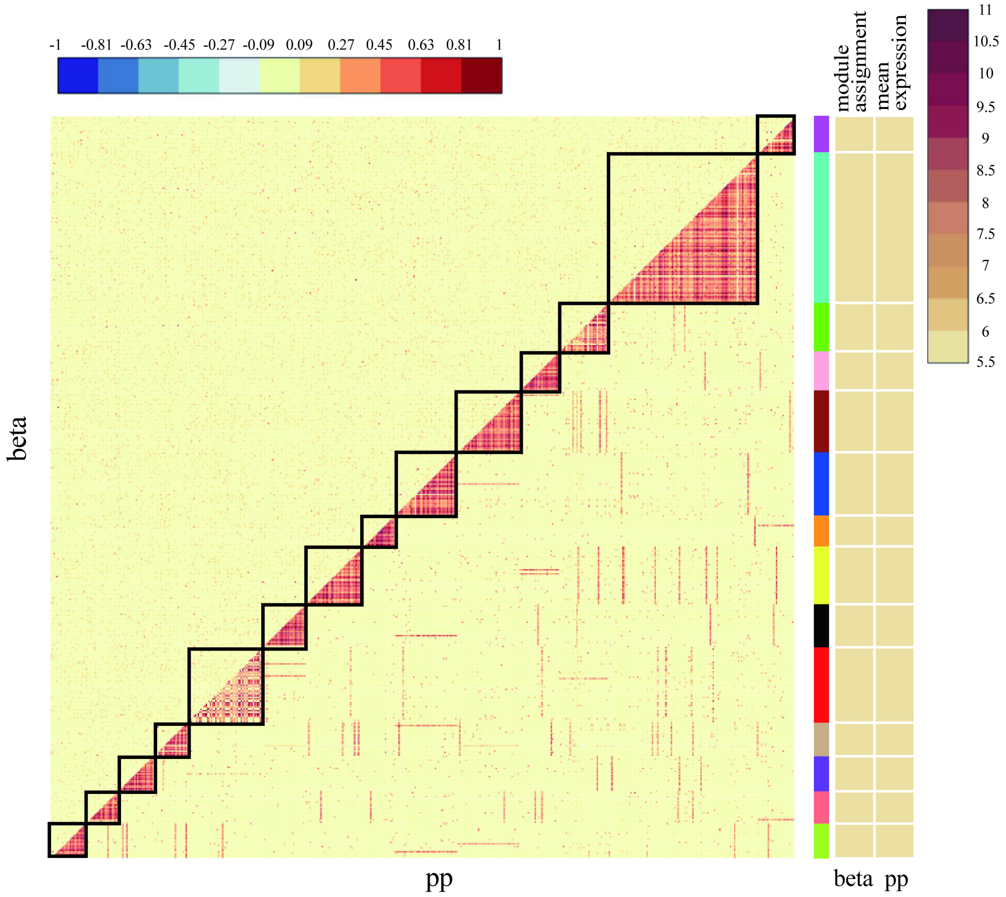

Supplementary Figure 23. Comparative correlation heat map of beta and pp. The upper diagonal of the main matrix shows a correlation between pairs of genes among the beta (the red color corresponds to positive correlations, blue to negative correlations). The lower diagonal of the heat map shows a correlation between the same gene pairs in the pp controls. Modules are identified in the heat map by black squares and on the right side of the heat map by a color bar. The brown bands on the right side indicate the mean expression of the modules in the beta (first column) and the pp (second column); darker colors indicate higher mean expression levels.

(11)delta-pp:

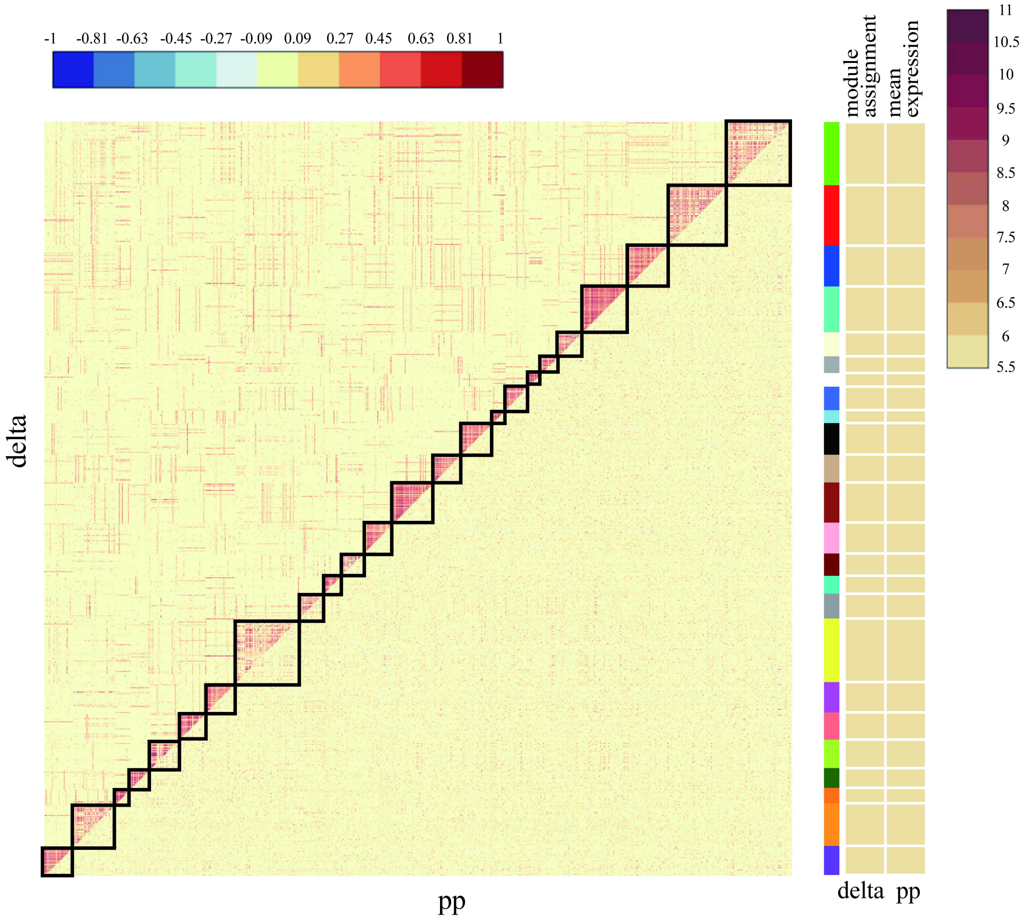

Supplementary Figure 24. Comparative correlation heat map of delta and pp. The upper diagonal of the main matrix shows a correlation between pairs of genes among the delta (the red color corresponds to positive correlations, blue to negative correlations). The lower diagonal of the heat map shows a correlation between the same gene pairs in the pp controls. Modules are identified in the heat map by black squares and on the right side of the heat map by a color bar. The brown bands on the right side indicate the mean expression of the modules in the delta (first column) and the pp (second column); darker colors indicate higher mean expression levels.
